# Electrical tuning of quantum light emitters in hBN for free space and telecom optical bands

**DOI:** 10.1038/s41598-024-51504-x

**Published:** 2024-01-08

**Authors:** Akbar Basha Dhu-al Shaik, Penchalaiah Palla, David Jenkins

**Affiliations:** 1 Department of Micro and Nanoelectronics, School of Electronics Engineering, Vellore Institute of Technology, Vellore, Tamil Nadu 632014 India; 2 School of Engineering, Computing and Mathematics, Faculty of Science and Engineering, University of Plymouth, Plymouth, England, UK

**Keywords:** Nanoscience and technology, Optics and photonics

## Abstract

Quantum light emitters (also known as single photon emitters) are known to be the heart of quantum information technologies. Irrespective of possessing ideal single photon emitter properties, quantum emitters in 2-D hBN defect structures, exhibit constrained quantum light emission within the 300–700 nm range. However, this emission range cannot fully satisfy the needs of an efficient quantum communication applications such as quantum key distribution (QKD), which demands the quantum light emission in fiber optic telecom wavelength bands (from 1260 to 1625 nm) and the free space optical (FSO) (UV-C-solar blind band—100 to 280 nm) wavelength ranges. Hence, there is a necessity to tune the quantum light emission into these two bands. However, the most promising technique to tune the quantum light emitters in hBN here, is still a matter of debate and till date there is no experimental and theoretical assurances. Hence, this work will focus on one of the most promising simple techniques known as Stark electrical tuning of the quantum light emission of hBN defect structures (N_B_V_N_, V_B_, C_B_, C_B_V_N_, C_B_C_N_, C_B_C_N_C_B_C_N_ complex, and V_B_O_2_). These hBN defects are designed and sandwiched as metal/graphene/hBN defect structure/graphene/metal heterostructure and electrically tuned towards FSO and fiber optic bands (tuning range from UV-C to O-band IR region) region, using constrained DFT computations. The external electric field predicted to yield an atomic bond angle tilt associated with this point defect structure creates out-of-plane dipole moments, enabling the tuning of quantum emission. This electrical tuning technique leads to a simple passive photonic component which enables easier compatibility with quantum circuits and it is found to be one of the perfect alternative solutions, which does not require much external hardware setup to implement as compared to earlier published strain induced tuning experiments.

## Introduction

Single photon sources are the essential building blocks in establishing optical quantum information technologies. For efficient quantum information applications like quantum communication (in particular, Quantum Key Distribution), it requires an integration of several quantum photonic circuits and devices, which are in-built with robust single photon emitters. This robustness should include quantum emission with high single photon purity, ability to with-stand at higher order elevated temperatures and more importantly, tunability towards necessary emission wavelengths (preferably, effortless tunable quantum emission). This tunability is necessary because, quantum applications like quantum key distribution (QKD) for long range distances via optical fibers^1–4^ demands single photon emission in telecom band (from 1260 to 1625 nm). Similarly, quantum communication for short distances (under non-line of sight (NLOS) condition) requires quantum emission in solar-blind (UV-C—100 to 280 nm) region^5,6^.

Another important aspect to be addressed is the feasibility and scalability of single photon emitters. In order to assemble a large-scale integrated quantum photonic circuit using quantum photonic chips, easily fabricated and scalable quantum emitters development is mandatory. These feasible and scalable quantum emitters should also be identical, which helps in fabricating a chip-scale array of quantum emitters.

Although bulk and three-dimensional single photon emitters exist, they have poor photon extraction efficiency and integration ability within photonic circuits. It is anticipated that the quantum emitters using layered materials (2D materials) will be one of the most promising solutions^7–11^. Of these, single photon emitters in 2-D hBN defect structure are found to exhibit robust quantum emission characteristics such as high single photon purity^12^, stable operation at higher order temperatures^13^ and external pressures^14^ etc..

At the leading edge of the research, carbon nanotubes can emit the single photons at higher wavelength (around 1500 nm), compared to 2-D hBN. But these nanotubes face the disadvantage of operating at low temperatures^15^.

However, quantum emitters in 2-D hBN defect structures faces the constraints of quantum emission bounded from 300 to 700 nm. But, very recent DFT predictions have claimed that, quantum emission from 2D hBN defect structures can also be tunable from telecom band to solar blind wavelength regions, by inducing externally controllable strains^16^ such as biaxial strains etc..

Even though this external strain inducement technique is effective in tuning the quantum emission towards necessary higher or lower order wavelengths, but exhibits the difficulties in fabricating the chip level quantum photonic circuits embedded with tunable single photon emitters. This is because, the quantum emitters needed to affix with a nano level cruciform structured polycarbonate (PC) beam^17^, to induce external strains (in order to tune the single photon emission), which is very complex task and is not much feasible. Moreover, fabricating the single photon emitting 2-D hBN layer in combination with this strain inducing cruciform PC structures, delaminates creating an array of single photon sources at chip level and further hinders the scaling process of quantum photonic circuits.

The only effective alternative solution to overcome this difficulty of tuning quantum emission, is to fabricate a tuning source which can be effortlessly embedded with quantum emitters to form integrated quantum photonic circuits. Such a quantum emission tuning source, may also be scaled up.

Till date quantum emission modulation, irrespective of external strain inducement technique^16,17^ is found be possible by inducing external field effects such as electric and magnetic fields.

**Figure 1:**
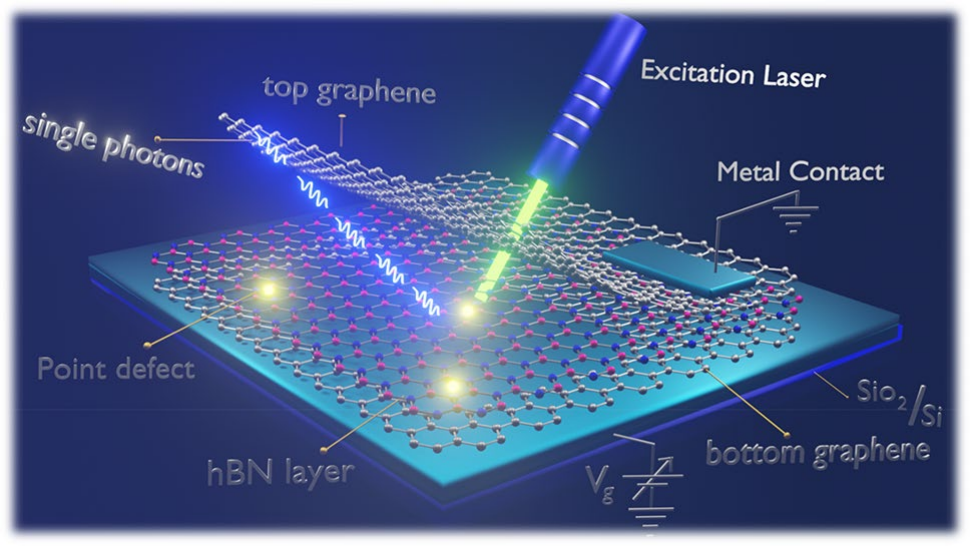
Schematic illustration of electrical tuning of quantum emitters (point defects) in hBN. The point defects engraved in monolayer hBN is sandwiched between graphene layers. The electric field is induced using metallic contacts and by varying the gate bias voltages, the quantum emission from point defects is found to be tunable.

From the output of our DFT computational research, we observed that tuning the quantum emission by inducing external electric field via Stark effect is an excellent alternative to the external strain inducement tunings. This electric field can be introduced at the vicinity of a quantum emitter, with the help of metallic gate electrodes. The tuning of quantum emitters in mono layer hBN defect structure by inducing external electric field through the gate electrodes is shown graphical abstract form in Fig. 1.

**Figure 2:**
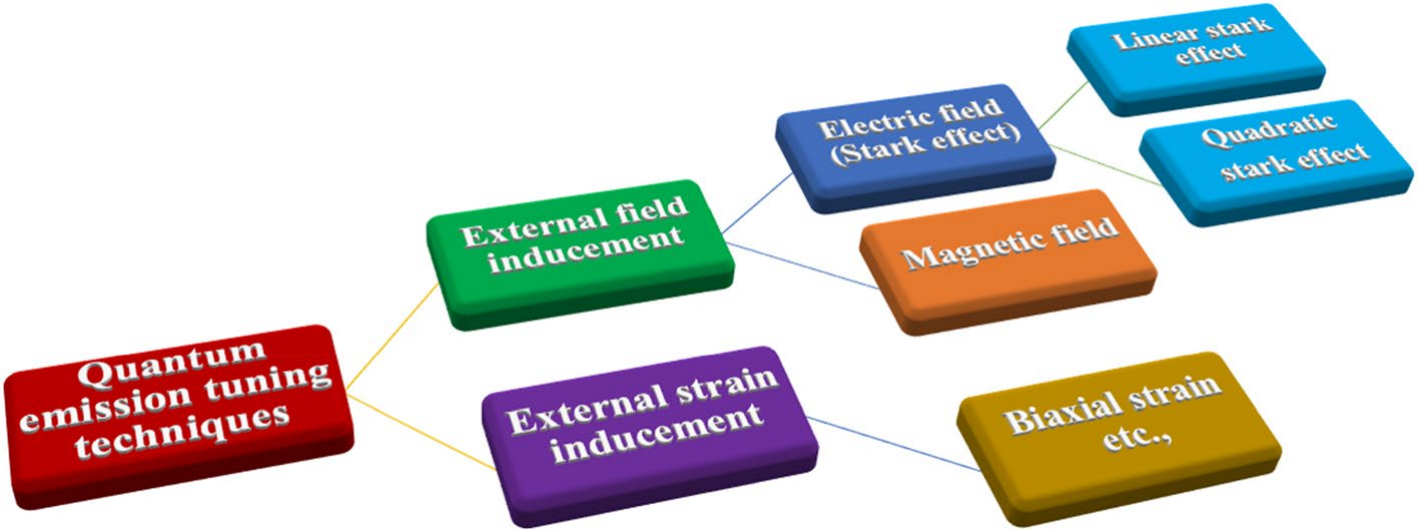
Pictorial representation of different possible techniques to tune the quantum emission from 2-D hBN defect structures.

According to data obtained from current quantum emission research literature, Fig. 2 shows a pictorial representation of different possible techniques to tune the quantum emission from 2-D hBN defect structures. Experimental research studies were also performed to alter the quantum emission in 2-D hBN by inducing external magnetic field^18,19^. But, tuning the quantum emission by inducing external magnetic field is out of our research interest.

As observed earlier, the electric field is found to be induced at the locality of quantum emitters in different ways. One preferable technique is, the luminescent point defect engraved monolayer hBN is sandwiched between graphene layers (which creates a van-der-Waals heterostructure). The metallic gate electrodes were positioned at the top and bottom of this van-der-Waals heterostructure^20,21,23^.

By applying a bias voltage across the gate electrodes, the electric field is induced at the vicinity of quantum emitters in hBN. These external electric fields creates a Stark effect, enabling tuning of quantum emission to take place.

Fabricating this kind of biasing hardware for quantum emission tuning, using metallic gate electrodes is very feasible than compared to other tuning techniques. These quantum emitter embedded 2-D heterostructures (graphene/hBN defect structure/graphene) can be easily incorporated into large scale integrated quantum photonic circuits, where tunable quantum emitters take a seat.

On top of that, this kind of feasible tuning platform can easily be scalable and promote the creation of an array of quantum emitters at chip level. Experimental studies were carried out to examine the electric field induced quantum emission tuning from 2-D hBN^20–23^, but the luminescent point defect structures, which are precisely responsible for this electrical quantum emission tuning is still a matter of debate, and to date there is no experimental and theoretical evidence, to determine the maximum tunability range of quantum emitters in 2-D hBN defect structures as a function of the applied electric field.

In this article, we examine the maximum possible quantum emission tunability range of 2-D hBN defect structures, by inducing an external electric field using constrained DFT computations. For this computation, we have chosen the precise luminescent point defects of three different types; intrinsic, extrinsic and passivated point defect structures. We have observed quantum emission tuning range from UV-C to O-band IR region by inducing an external electric field. This electric field induced tuning technique is found to be more feasible to fabricate and can be easily embedded into large scale integrated quantum photonic circuits. Also, this technique can efficiently support high scalability of these sources in order to create an array of tunable quantum emitters enabling quantum photonic circuits, which may lead many efficient quantum information technology applications.

## Materials and methodology

Here, we mainly focus on tuning the quantum emission by inducing external electric field using DFT computations. This electric field induced tuning phenomena is called electrical quantum emission tuning via the Stark effect.

### Stark effect

The shifting of spectral lines of atoms and molecules under the presence of an external electric field is called the Stark effect.

Here, shifting of energy states by inducing electric field leads to quantum emission tuning. To elucidate, luminescent point defect structures create intermediate energy states, in between the wide band gap energy of 2-D hBN. Therefore, electron transition and relaxation between this intermediate energy states leads to quantum emission. Hence, by inducing a strong electric field, this intermediate energy w.r.t its mean position states can be controlled, which can increase or decrease the band gap energy. This increase and decrease in energy gaps lead to tuning the quantum emission. This Stark effect is majorly classified into two types. They are:

1. ***Linear Stark effect*** This linear Stark effect, arises for a naturally occurring non-symmetric distribution of electrical charges. The electric field strength will be typically low in this effect. Most of the times this linear Stark effect is observed for hydrogen and hydrogen like low-electron atoms.
2. ***Quadratic Stark effect ***This quadratic Stark effect, arises when an external high electric field is induced. This kind of Stark effect occurs when, an external electric field exceeds around 10^7^ V/m. This quadratic Stark effect is mostly observed at atoms with many-electrons. Here in this quantum emission tuning from 2-D hBN defect structure, inducing higher external electric field will be creating a quadratic Stark field effect.

### Initial optimization and elementary parametric considerations to be employed before simulating electric field inducement to the quantum emitters

A well optimized hBN layer with associated defect structures were adopted for this DFT computations. The defective hBN monolayers were created using a 7 × 7 supercell. The geometrical optimizations were performed using Monkhurst-Pack reciprocal space grid. The linear combination of atomic orbital (LCAO) calculations was utilized for these simulations. This LCAO processes the atomic simulations, by employing Generalized Gradient Approximation (GGA) to the exchange correlation functional proposed by Perdew, Burke and Ernzerhof (PBE)^24^. The nucleus–electron interaction is represented by Fritz-Haber Institute (FHI) pseudopotentials.

These pseudopotentials computes according to the methodology reported by Troullier and Martins^25^. A Gaussian smearing occupation is employed for numerical accuracy, with an energy tolerance of 0.01 eV. All the calculations are spin-polarized. We employed two different basis sets i.e., Tight Tier-1 and Double-zeta plus polarization (DZP), for different defect structures in order to analyse the computational efficiency comparisons and we observed that both of the basis sets are almost equally efficient, particularly in this kind of tuning the quantum emission simulations.

This sort of LCAO calculations, similar basis sets and pseudopotentials were concern from earlier strain inducement quantum emission tuning^16^ DFT computations.

### Creating the Stark effect at the 2-D hBN quantum light emitters

By using DFT computations we have observed the effect of external electric field on different quantum emitters in 2-D hBN defect structures. We have observed the tuning of zero phonon lines (ZPL) quantum emission from the point defects via Stark effect. By implementing this Stark effect, an electric field is introduced at vicinity of point defect structures and so that ZPL quantum emission energy of point defects found to be altered as observed in ref^20–23^.

Experimentally, the electric field is induced at the quantum emitters byinducing a vertical electric field (using top and bottom electrodes) to the luminescent point defects engraved hBN monolayer and this point defected 2D-hBN is sandwiched between graphene layers (creates van-der-waals heterostructure)^20,21,23^. Hence, by applying metallic gate electrode voltages, an electric field is induced and leads to tuning of quantum emission.

The graphene layers are just used for providing very low electrical contact resistance at the interface of 2-D hBN defect structure and metallic electrodes, because high electrical contact resistance can cause substantial heating and also leads to damage of devices.

**Figure 3:**
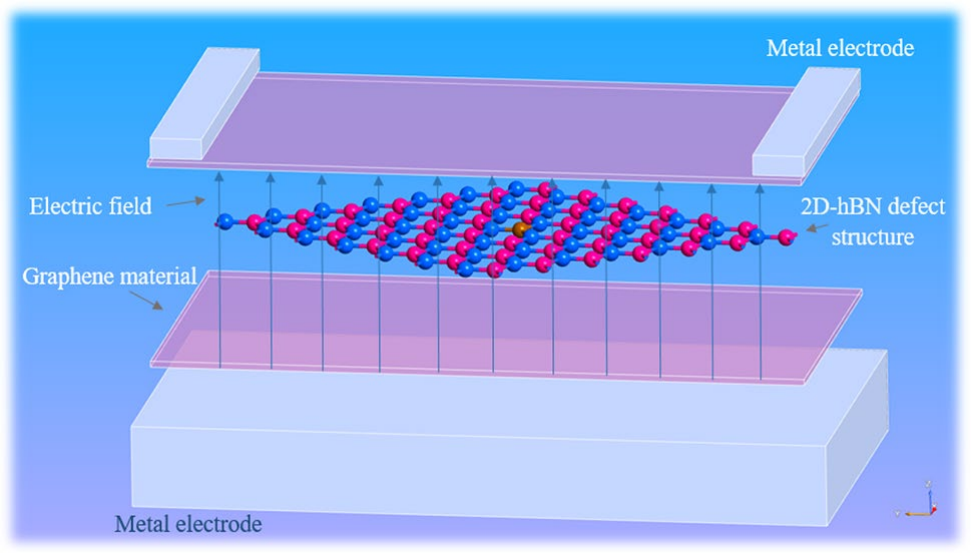
Schematic illustration of electric field inducement at the defect embedded hBN monolayer by using metallic gates. Inducing a vertical electric field to the luminescent point defect engraved hBN layer, by coupling with top and bottom metallic gates. The bias voltage will be applied at one of the electrodes and assigning the other metal electrode to ground as shown in Fig. 1. The defected hBN layer was sandwiched with top and bottom graphene material (assigned with relative permittivity of graphene = 6.9ε_0_) and then the electric field was induced with metallic gate electrodes.

Hence, by make using advantage of the DFT computational tool alleviation, we have introduced the possession of graphene layers (top and bottom of defected 2D hBN) in electrical tuning of quantum emission from defects. We sandwiched the point defected 2D hBN with a material whose relative permittivity = 6.9ε_0_ (graphene monolayer’s relative permittivity) as shown in Fig. 3 and we examined the emission tunability of quantum emitters, by electric field inducement using metallic gates. The computational schematic shown in Fig. 3 is an integral part of experimental device design structure which is already depicted in Fig. 1.

In earlier experiments of tuning the quantum emission via Stark effect, an electric field strength around 0.0005 K V/nm was induced^20^. However, in our DFT computations, we induced a higher electric field strength than the above experimental quantities, in order to examine the maximum tunability of quantum emitters via Stark effect.

Once after proper optimization of defective hBN monolayer, the external electric field is induced by introducing top and bottom metallic gate electrodes to defected 2D hBN (as shown in Fig. 3). By using multi-grid Poisson solvers in this DFT computations, dirichlet boundary conditions were assigned for both the gate electrodes. This boundary conditions helps in allocating static electric field inducing directions towards hBN monolayer.

### Interpreted data for analysis

To showcase, compare and analyze the quantum emission tunability of luminescent point defects using Stark effect, we extracted necessary computed, analytic data such as optical emission spectrum of point defect structures, band structures and their corresponding projected density of states (PDOS) etc., by performing this constrained DFT simulations.

#### Optical emission spectrum

The optical emission spectrum of luminescent point defect structures found to exhibit Lorentzian shaped sharp isolated emission peaks at their corresponding energies. The reason behind this sharp isolated emission peak is that, as per earlier references^26^, these luminescent point defects forms an intermediate energy levels (an electron occupied ground state and un-occupied excited state) between valence and conduction bands of 2D hBN.

This occupied ground state electron transmits to un-occupied excited state, if it excites with enough energy. This single electron transition, emits a single photon of specific wavelength while relaxing back to ground state. The DFT computations addresses this single photon emission by a Lorentzian shape peak (sharp emission peak), and the quantum studies notify this sharp emission peak as a zero-phonon line (ZPL).

Here we provide the list of various luminescent point defect structures and their corresponding ZPL energies in Table 1, measured after enough optimization of defective hBN layers with zero induced static electric fields. We also recorded the shifts in ZPL energies after applying different gate voltages (verifying at different electric field strengths).

**Table 1:** ZPL energies of point defects without any tuning.

Sl. no	Point defect	ZPL energies (DFT simulated)
1.	N_B_V_N_	2.2 eV
2.	V_B_	4.32 eV
3.	C_B_V_N_	1.38 eV
4.	C_B_	1.56 eV
5.	C_B_C_N_	3.66 eV
6.	C_B_C_N_C_B_C_N_ type-1	1.86 eV
7.	C_B_C_N_C_B_C_N_ type-2	
8.	VBO_2_	1.86 eV

A minute variations in ZPL energies (shown in Table 1), were observed compared to earlier experimental and DFT computed values, this is because of the influence of multiple DFT constraints such as general optimization conditions, Poisson solvers dirichlet boundary conditions etc.

#### Band structures and PDOS

The band structures and PDOS exhibit the projected graphical illustration of electron transition possibilities due to energy gaps in between the intermediate energy states (electron occupied and un-occupied energy states) occurred due to the luminescent point defect structures. These single electron transition and relaxation leads to single photon emission. So that the energy difference between these occupied and un-occupied states (measured in band structures and PDOS) must be consistent with ZPL energies (measured in optical emission spectrum) of luminescent point defect structures. This consistency confirms the nature of quantum emission from point defects.

We also recorded the modulated band structures and PDOS plots of defect structures, before and after inducement of electric fields. These DFT simulations were performed using Synopsys QuantumATK Q-2019.12-SP1 software package (atomic-scale modelling software)^27^.

## Results and discussion

The defect structures chosen in this work are listed in Table 1. The reason behind adopting the above listed point defects, for electrical tuning of quantum light emission via Stark effect is, most of these published defects’ structures already found to exhibit greater quantum emission tuning by inducing external strain gradients techniques as observed in ref^16,28,29^. Also, these defect structures are found to be easily fabricated, by using simple fabrication techniques as in ref^26,30–36^.

**Figure 4:**
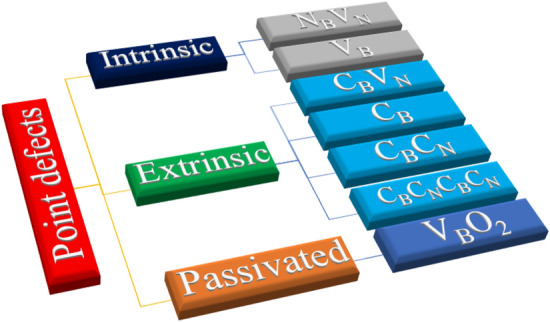
Pictorial representation of classification of 2-D hBN quantum light emitters.

We have broadly classified these selectively chosen point defect structures into three categories as illustrated in Fig. 4.

### Wavelength tuning of quantum light emitters via Stark effect

#### Stark tuning of intrinsic point defect structures

As listed in Fig. 4, two-point defects were considered under intrinsic defect structures category. They are:

- Nitrogen mono vacancy with self-interstitial (N_B_V_N_) defect
- Boron mono vacancy (V_B_)

##### Nitrogen mono vacancy with self-interstitial (N_B_V_N_)

Among numerous defect structures predicted to-date, nitrogen mono vacancy with self-interstitial (boron replaced by nitrogen) (N_B_V_N_), is the only point defect, whose DFT approximations are closely compatible with several experimental observations and also N_B_V_N_ defect is the mostly referred luminescent defect structure till date. This N_B_V_N_ defect can be easily incorporated in layered hBN by simple annealing at 850 °C under 1 torr of argon for 30 min^26^.

**Figure 5:**
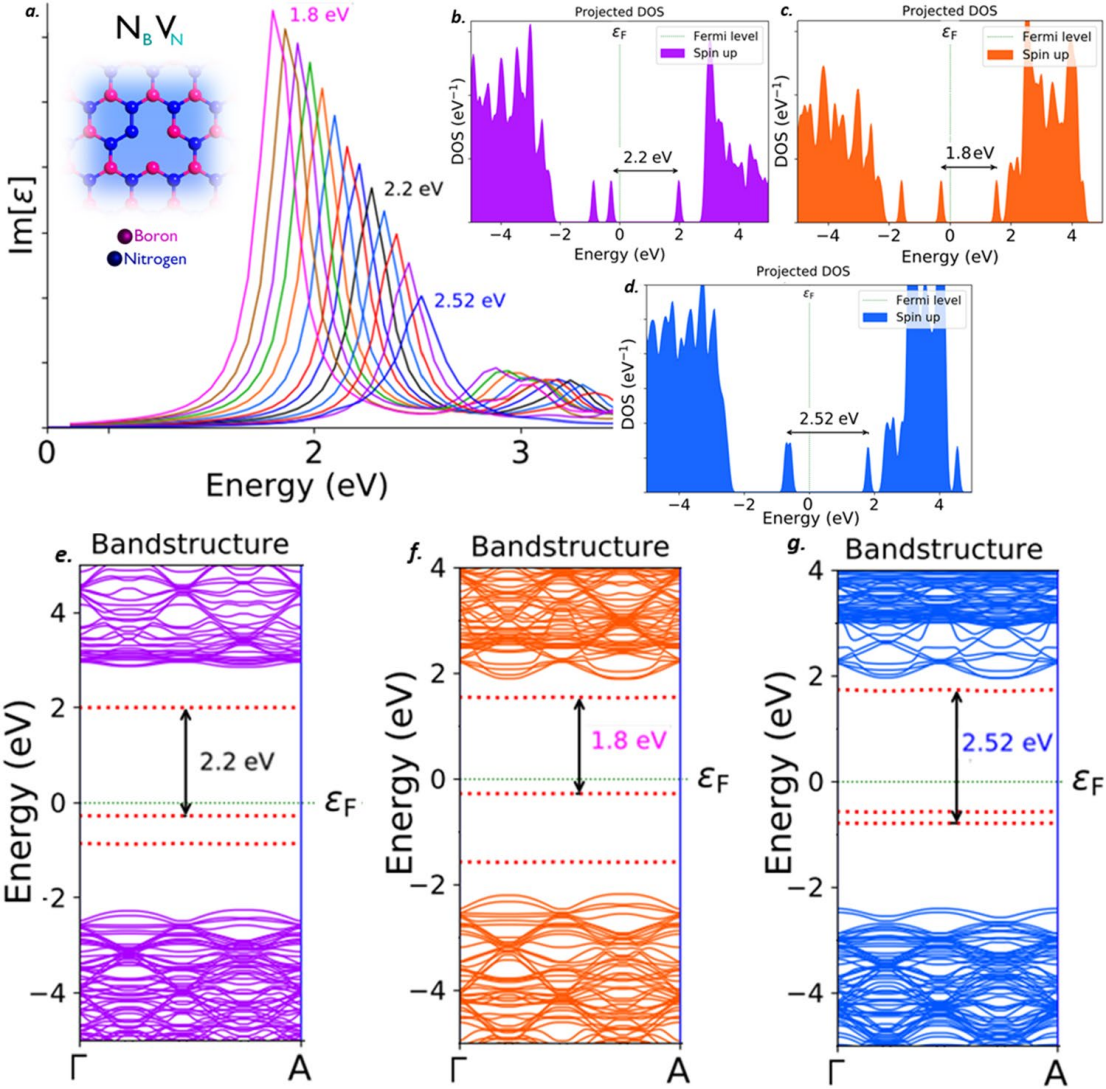
Schematic illustration of N_B_V_N_ defect, its complete optical emission spectrum (tuned by external electric field inducement), corresponding PDOS and band structures. (**a**) The optical emission spectrum with maximum possible tunability due to electric field inducement and the inset cartoon shows the schematic of N_B_V_N_ defect. The ZPL emission without any electric field applied is observed at around 2.2 eV (black curve). (**b**) Corresponding PDOS of N_B_V_N_ defect engraved hBN layer without inducing electric field. (**e**) Corresponding energy band structure plot for zero electric field induced, defected hBN layer, whose energy difference is consistent with ZPL and PDOS (Fig. 5(b)). (**c**) Corresponding PDOS of negative electric field induced N_B_V_N_ defected hBN layer. (**f**) Corresponding energy band structure plot for negative electric field inducement, whose reduced energy gap between intermediate states is consistent with tuned ZPL and PDOS (Fig. 5(**c**)). (**d**) Corresponding PDOS of positive electric field induced N_B_V_N_ defected hBN layer. (**g**) Corresponding energy band structure plot for positive electric field inducement, whose increased energy gap between intermediate states is consistent with tuned ZPL and PDOS (Fig. 5(**d**)). Optical emission spectrum plots were extracted by assigning y-axis to imaginary component of dielectric constant [ε] and x-axis to energy (eV). In all the energy band structure plots intermediate states were highlighted with red dotted lines.

The N_B_V_N_ defect found to emit single photons in visible region. This N_B_V_N_ defect exhibits a sharp Lorentzian shaped zero phonon line (ZPL) emission peak around 2.2 eV (black curve) as shown in Fig. 5a, without inducing any tuning biases like external deformative strain and electric field etc. The projected density of states (PDOS) of N_B_V_N_ defect under no external static electric field inducement is shown in Fig. 5b.

The PDOS exhibits the formation of new electron occupied and un-occupied intermediate energy states (formed due to N_B_V_N_ defect), which are separated by a Fermi level in between. The energy difference between these intermediate energy states is consistent with ZPL emission energy. The ZPL is the energy difference between the excited state and ground state. Similar kind of virtual appearance of intermediate energy states (formed due to N_B_V_N_ defect), can also be analysed by energy band structure of N_B_V_N_ defect engraved hBN layer, as shown in Fig. 5e. This energy band structure plot was extracted for defect induced hBN layer, without any deformative external strain and electrical tuning biases etc., All the intermediate energy states projected in PDOS, can also be observed in energy band structure plots (highlighted by red dotted lines). The energy gap between these intermediate energy states highlighted in band diagram are also consistent with values of PDOS and ZPL energies. The electron transition and spontaneous relaxation between these intermediate energy states leads to single photon emission in visible region around 2.2 eV.

Now we have induced electric field through metallic gates to the N_B_V_N_ defect engraved hBN layer. By applying a negative gate voltage at the metallic electrode, we observed the quantum energy tuning towards lower energy region and vice versa for positive gate voltage, the quantum energy tuning towards higher energy region were observed. In order to examine the defect’s extreme tunability, we have induced higher order gate voltages and we observed the tunability towards lower energy region around 1.8 eV (for higher negative gate voltage) and the tunability towards higher energy region around 2.52 eV (for higher positive gate voltage). The complete tunability of N_B_V_N_ defect for the applied electric field was shown in Fig. 5a and the magnitude of electric field applied for this greater tunability was listed in Table 2.

**Table 2:** Complete tunability range of different luminescent point defects in 2D hBN due to electric field inducement and the corresponding electric field strength.

Point defect	Tunability observed towards lower energy region and responsible electric field strength		Tunability observed towards higher energy region and responsible electric field strength	
	Tunability range observed towards lower energy region	Electric field strength (K V/nm)	Tunability range observed towards higher energy region	Electric field strength (K V/nm)
N_B_V_N_	2.2–1.8 eV	− 0.054	2.2–2.52 eV	0.048
V_B_	4.32–3.84 eV	0.059	4.32–4.68 eV	− 0.059
C_B_V_N_	1.38–0.96 eV	0.063	1.38–1.5 eV	− 0.04
C_B_	1.56–1.08 eV	− 0.076	1.56–1.8 eV	0.059
C_B_C_N_	3.66–3.18 eV	− 0.08	3.66–3.72 eV	0.03
C_B_C_N_C_B_C_N_ type-1	1.86–1.8 eV	0.04	1.86–2.04 eV	− 0.081
C_B_C_N_C_B_C_N_ type-2	1.86–1.68 eV	0.057	1.86–1.98 eV	− 0.034
VBO_2_	–	–	1.86–2.16 eV	0.08

We also examined the PDOS and energy band structure plots of electric field induced defective hBN layers. The decrease in energy gaps is due to de-escalation of intermediate energy states for the higher order negative gate voltage was shown in Fig. 5c and the similar behaviour was observed in band structure plots as shown in Fig. 5f. The diminished energy gap in PDOS and band structure plots were consistent with tuned ZPL energy (around 1.8 eV), towards lower energy region. This consistency confirms the quantum emission tuning towards lower energy region, for the electric field induced and magnitude of electric field applied for tuning towards lower energy region was listed in Table 2.

Similarly, for higher order positive gate voltages, the energy gaps between this intermediate energy states were inclined and this inclination of energy gaps in PDOS and band structure plots were as shown in Fig. 5d and g respectively. The increased energy gap values of PDOS and energy band structure were consistent with tuned ZPL energy (around 2.52 eV), towards higher energy region. This consistency assures the quantum emission tuning towards higher energy region for the applied electric field.

The complete tunability of N_B_V_N_ defect, is observed in visible region and the tunability emission range was listed in Table 3. This kind of quantum emission tunability towards broad visible region enhances the implementation of small-scale quantum photonic devices, quantum sensors and quantum lamps etc.

**Table 3:** Complete emission tunable region and tunability range of all the different luminescent point defects in 2D hBN and their corresponding optical spectrums.

Point defect	Complete tunable emission range and corresponding optical emission spectrum graphs		Tunable emission region
	Complete tunable emission range observed	Optical spectrum graphs (details)	
N_B_V_N_	1.8–2.52 eV	Fig. 5	Visible region
V_B_	3.84–4.68 eV	Fig. 7	UV-C to UV-A
C_B_V_N_	0.96–1.5 eV	Fig. 8	Near-IR region
C_B_	1.08–1.8 eV	Fig. 9	Visible-Near-IR
C_B_C_N_	3.18–3.72 eV	Fig. 10	UV-A region
C_B_C_N_C_B_C_N_ type-1	1.8–2.04 eV	Fig. 11	Visible region
C_B_C_N_C_B_C_N_ type-2	1.68–1.98 eV	Fig. 12	Visible region
VBO_2_	1.86–2.16 eV	Fig. 13	Visible region

### Physical interpretation of quantum light emission wavelength shifts due to electric field inducement for N_B_V_N_ defect

We also interpreted the main reason behind this wavelength shifts in quantum emission, due to electric field inducement. As per earlier DFT literature^20^, external electric field tilts the atomic bond angles in defect structure and this leads to popping out a portion of defect structure towards out-of-the crystal plane. This creates a dipole moments at out-of-plane environment. This transpose of dipole towards out-of-plane condition due to varying external electric field strength, causes shifting and tuning of quantum emission.

To examine this hypothesis, we have chosen two precise point defect structures (N_B_V_N_ and C_B_V_N_), which are mostly referred by first principle DFT studies^26,31^. By using the contrived DFT computations, we intentionally introduced the different atomic bond angle tilts, which leads to protrude a chunk of defect structure towards out-of-the crystal plane with different displacements (Δ). Interestingly, due to this atomic displacements we observed the shifts in quantum emission.

**Figure 6:**
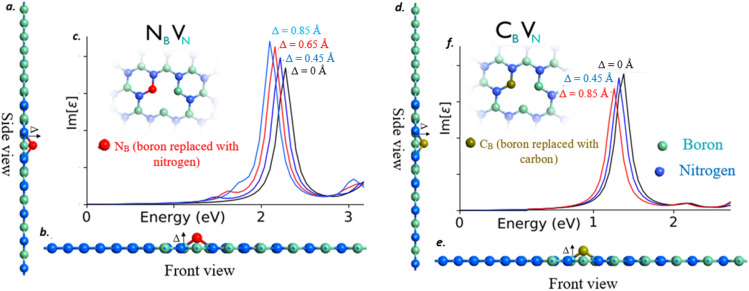
side and front view of N_B_V_N_ and C_B_V_N_ defects engraved monolayer hBN and corresponding shifts in quantum emission. (**a**, **b**) side and front view of monolayer hBN in which N_B_V_N_ defect is engraved. N_B_ chunk from N_B_V_N_ defect is protruded towards out-of-the crystal plane due to atomic bond angle tilts. (**c**) Corresponding shifts in quantum emission for different atomic displacements. This different atomic displacements (Δ) were created due to different atomic bond angle tilts. Inset of the Fig. 6(**c**) shows the N_B_V_N_ defect structure in which N_B_ segment is popped out. (**d**, **e**) side and front view of monolayer hBN in which C_B_V_N_ defect is engraved. C_B_ chunk from C_B_V_N_ defect is protruded towards out-of-the crystal plane due to atomic bond angle tilts. (**f**) Corresponding shifts in quantum emission for different atomic displacements. This different atomic displacements were created due to different atomic bond angle tilts. Inset of the Fig. 6(**f**) shows the C_B_V_N_ defect structure in which C_B_ segment is popped out.

Figure 6a,b represents the side and front view of N_B_V_N_ defect engraved monolayer hBN, in which N_B_ chunk from N_B_V_N_ defect is popping out-of-crystal plane. Figure 6c represents the corresponding shifts in quantum emission for different atomic displacements (Δ) of N_B_ segment from N_B_V_N_ defect. Particularly, the computational results shown in Fig. 6 are recorded without inducing any external electric fields.

These shifted quantum emissions from N_B_V_N_ (due to out-of-plane atomic displacement as shown in Fig. 6) were consistent and almost similar to the tuning observed due to electric field inducement as shown in Fig. 5a. This consistency gives an assurance to confirm the hypothesis of external electric field creates a dipole moment towards out-of-the crystal plane due to atomic bond angle tilt.

#### Boron mono vacancy (V_B_)

From the list of luminescent point defect structures, only Boron mono vacancy (V_B_) defect structure exhibits the quantum emission in UV region, which helps in tuning the quantum emission towards deep UV region and makes an advantage of implementing robust quantum communication in UV region for short range services^5,6^. Boron mono vacancies are more preferably formed by controlled electron beam irradiation with energy of 120 keV, through a layer-by-layer sputtering process^36^.

**Figure 7:**
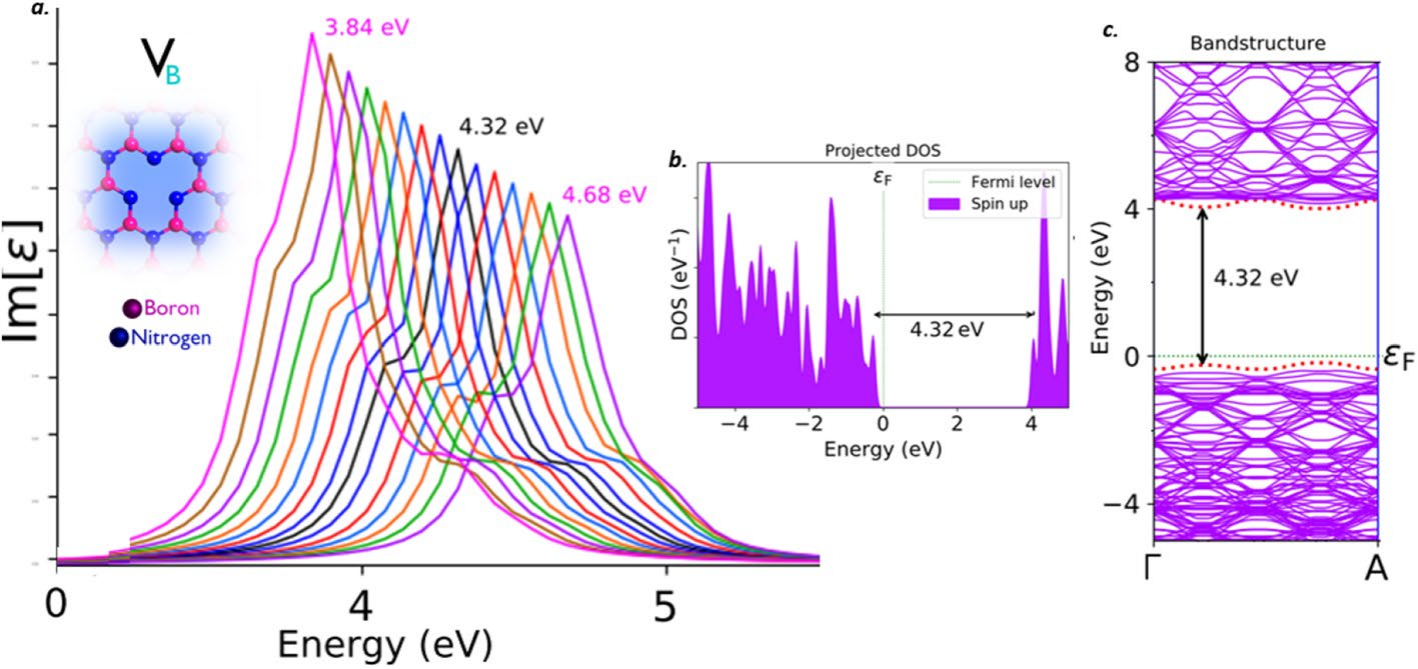
Schematic illustration of V_B_ defect, its complete optical emission spectrum (tuned by external electric field inducement), corresponding PDOS and band structures. (**a**) The optical emission spectrum with maximum possible tunability due to electric field inducement and the inset cartoon shows the schematic of V_B_ defect. The ZPL emission without any electric field applied is observed at around 4.32 eV (black curve). (**b**) Corresponding PDOS of V_B_ defect engraved hBN layer without inducing electric field. (**c**) Corresponding energy band structure plot for zero electric field induced, defected hBN layer, whose energy difference is consistent with ZPL and PDOS (Fig. 7(**b**)).

The V_B_ defect, found to emit the single photons around the frontier of UV-B and UV-C region. This V_B_ defect found to exhibit a Lorentzian shaped ZPL sharp emission peak around 4.32 eV (black curve), as shown in Fig. 7a, without inducing any external tuning biases like external deformative strain and electric field inducement etc. The PDOS of V_B_ defect under no external static electric field inducement is shown in Fig. 7b, which represents the formation of new electron occupied and un-occupied intermediate energy states (formed due to V_B_ defect) and separated by a Fermi level in between. The energy difference between these intermediate energy states is found to be consistent with ZPL emission energy.

Similar virtual appearance of intermediate energy states (formed due to V_B_ defect), can also be analyzed by energy band structure of V_B_ defect incorporated in hBN layer, as shown in Fig. 7c. This energy band diagram was obtained, without inducing any deformative external strain and electrical tuning biases etc., to the defective hBN layer. The projected intermediate energy states in PDOS, also reflected in energy band structure plots (highlighted by red dotted lines). Thus, the energy gap between these intermediate energy states highlighted in band diagram and the energy states projected in PDOS are consistent with each other and also consistent with ZPL energy values. The electron transition between these intermediate energy states and further spontaneous emission leads to single photon emission in UV region around 4.32 eV.

The complete tunability of V_B_ defect, is observed from UV-A to UV-C region and the tunability emission range was listed in Tables 2 and 3. The detailed explanation related to electric field inducement to the V_B_ defected hBN layer was provided in supporting information (Section-1). This kind of quantum photonic circuit level, electrical tuning of quantum emission towards deep UV-C region, efficiently enhances the development of quantum communication in UV region.

#### Stark tuning of extrinsic point defect structures

Out of several extrinsic defects, we mainly focus on carbon related defects, because these carbon based luminescent point defects proven to be one of the efficient single photon sources in many experimental and first principle DFT predictions. Among numerous carbon based defect structures, we selected a few point defects, which are mostly referred and have more favourable forming conditions. Such defect structures are:

- Nitrogen mono vacancy with carbon-interstitial (C_B_V_N_),
- Single boron substitutional with carbon (C_B_),
- Single boron and single nitrogen substitutional with carbons (C_B_C_N_) and Carbon dimer complex (C_B_C_N_ C_B_C_N_).

##### Nitrogen mono vacancy with carbon-interstitial (C_B_V_N_)

In this list of carbon based defects, the defect structure with high favourable formation conditions after C_B_ defect and highly addressed defect structure after N_B_V_N_ defect, is Nitrogen mono vacancy with carbon-interstitial (C_B_V_N_) defect. This C_B_V_N_ defect originates by controlled incorporation of impurities (carbon) via efficient bottom-up synthesis methods like MOVPE, MBE and HOPG or controlled carbon ion implantation at an energy of 10 keV^31^.

**Figure 8:**
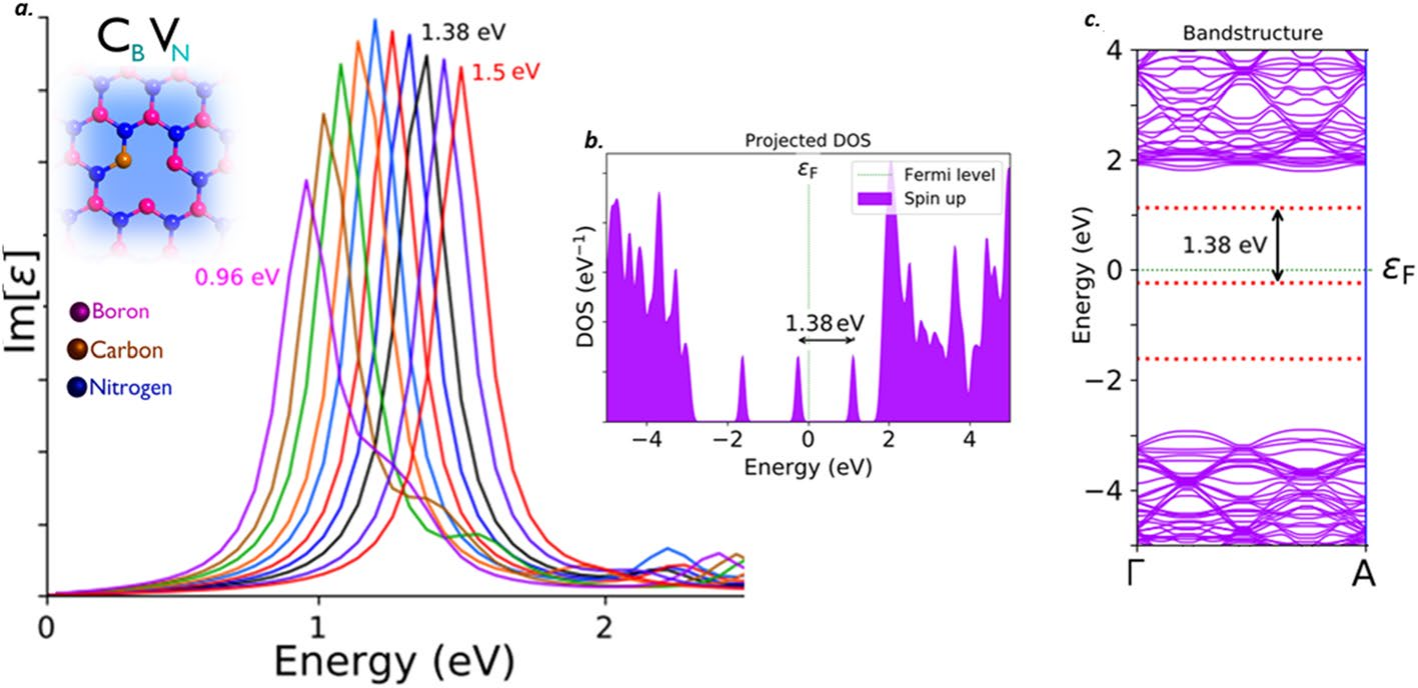
Schematic illustration of C_B_V_N_ defect, its complete optical emission spectrum (tuned by external electric field inducement), corresponding PDOS and band structures. (**a**) The optical emission spectrum with maximum possible tunability due to electric field inducement and the inset cartoon shows the schematic of C_B_V_N_ defect. The ZPL emission without any electric field applied is observed at around 1.38 eV (black curve). (**b**) Corresponding PDOS of C_B_V_N_ defect engraved hBN layer without inducing electric field. (**c**) Corresponding energy band structure plot for zero electric field induced, defected hBN layer, whose energy difference is consistent with ZPL and PDOS (Fig. 8(**b**)).

The C_B_V_N_ defect, found to emit the single photons around the frontier of near-IR region. This C_B_V_N_ defect exhibits a ZPL emission sharp Lorentzian shaped peak around 1.38 eV (black curve) as shown in Fig. 8a, without any tuning biases inducement like external deformative strain and electric field etc.,. The graphically represented PDOS and similar virtually analytic energy band structure plot of C_B_V_N_ defect under no external static electric field inducement were shown in Fig. 8b and c respectively, which represents the formation of new electron occupied and un- occupied intermediate energy states (formed due to C_B_V_N_ defect) and separated by a Fermi level in between. The projected intermediate energy states in PDOS, also observed in energy band structure plots (highlighted by red dotted lines). The energy difference between these intermediate energy states in PDOS and band structure are found to be consistent with each other and also consistent with ZPL emission energy. The electronic transition and followed by spontaneous emission between these intermediate energy states, leads to single photon emission around near IR region at 1.38 eV.

The complete tunability of C_B_V_N_ defect, is observed in deep near-IR region and the tunability emission range was listed in Tables 2 and 3. The detailed explanation related to electric field inducement to the C_B_V_N_ defected hBN layer was provided in supporting information (Section-2). This kind of electrical tuning of quantum emission at O-band IR region, efficiently enhances the fabrication of quantum photonic circuits and implementation of robust quantum communication which finds application in quantum cryptographic technologies.

### Physical interpretation of quantum light emission wavelength shifts due to electric field inducement for C_B_V_N_ defect

The main reason behind this wavelength shifts in C_B_V_N_ quantum emission, due to electric field inducement is external electric field tilts the atomic bond angles in defect structure and this leads to create an out-of-plane dipole moment. This dipole towards out-of-plane dipole causes shifting and tuning of quantum emission.

Figure 6d,e represents the side and front view of C_B_V_N_ defect engraved monolayer hBN, in which C_B_ chunk from C_B_V_N_ defect is popping out-of-crystal plane. Figure 6f represents the corresponding shifts in quantum emission for different atomic displacements (Δ) of C_B_ segment from C_B_V_N_ defect. Particularly, the computational results shown in Fig. 6 are recorded without inducing any external electric fields.

These shifted quantum emissions from C_B_V_N_ defect (due to out-of-plane atomic displacement as shown in Fig. 6) were consistent and almost similar to the tuning observed due to electric field inducement as shown in Fig. 8a respectively. This consistency gives an assurance to confirm the hypothesis of external electric field creates a dipole moment towards out-of-the crystal plane due to atomic bond angle tilt.

#### Single boron substitutional with carbon (C_B_)

Among several carbon related defect structures, single boron atom substitutional with carbon (C_B_), was the only defect with high feasibility of formation, when irradiated with carbon atoms.

**Figure 9:**
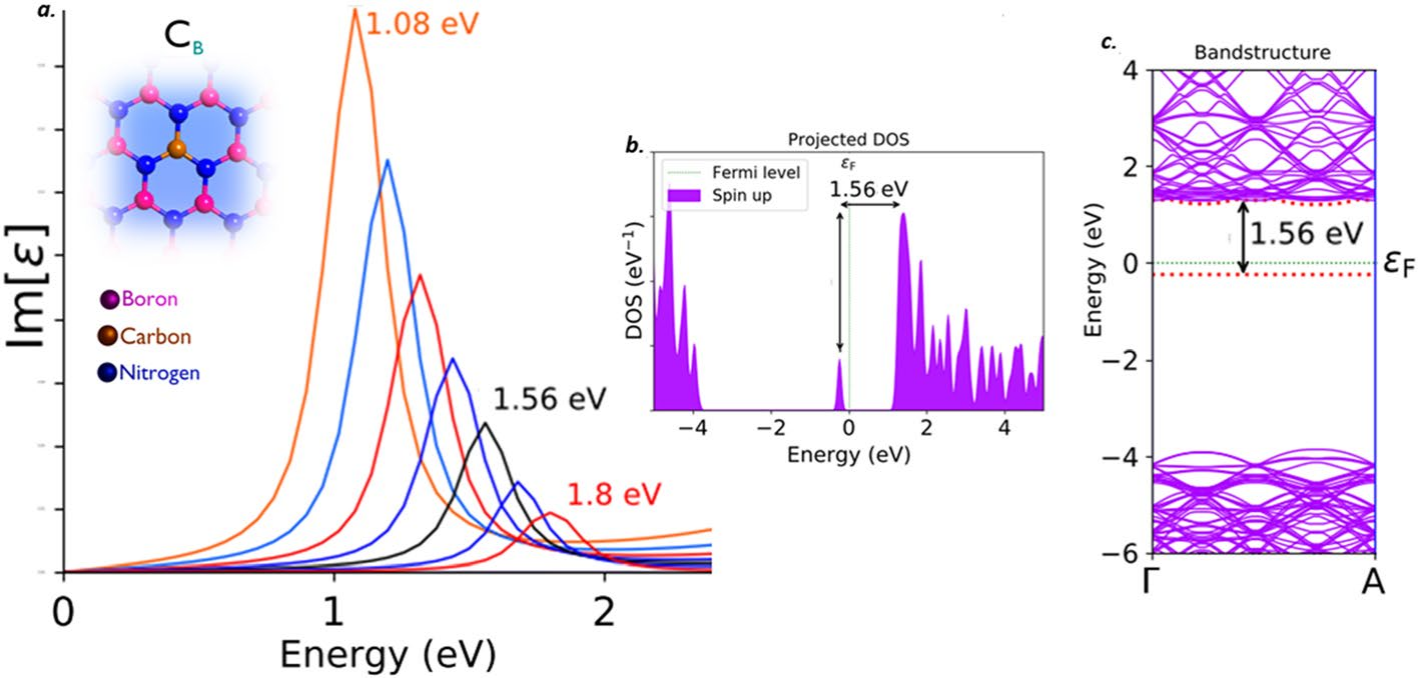
Schematic illustration of C_B_ defect, its complete optical emission spectrum (tuned by external electric field inducement), corresponding PDOS and band structures. (**a**) The optical emission spectrum with maximum possible tunability due to electric field inducement and the inset cartoon shows the schematic of C_B_ defect. The ZPL emission without any electric field applied is observed at around 1.56 eV (black curve). (**b**) Corresponding PDOS of C_B_ defect engraved hBN layer without inducing electric field. (**c**) Corresponding energy band structure plot for zero electric field induced, defected hBN layer, whose energy difference is consistent with ZPL and PDOS (Fig. 9(**b**)).

This C_B_ defect found to emit single photon around the boundary of near IR region^37,38^. This C_B_ defect exhibits a Lorentzian shaped ZPL sharp emission peak around 1.56 eV (black curve) as shown in Fig. 9a, without any tuning biases inducement like external deformative strain and electric field etc.,. The graphically represented PDOS of C_B_ defect under no external static electric field inducement is shown in Fig. 9b. The PDOS exhibits the formation of new electron occupied and un-occupied intermediate energy states (formed due to C_B_ defect), which are separated by a Fermi level in between.

The energy difference between these intermediate energy states is consistent with ZPL emission energy. Similar kind of virtual appearance of intermediate energy states (formed due to C_B_ defect), can also be analyzed by energy band structure of C_B_ defect engraved hBN layer, as shown in Fig. 9c. This energy band structure plot was extracted for defect induced hBN layer, without any deformative external strain and electrical tuning biases etc., All the intermediate energy states projected in PDOS, can also be observed in energy band structure plots (highlighted by red dotted lines).

The energy gap between these intermediate energy states highlighted in band diagram are also consistent with values of PDOS and ZPL energies. The electron transition and spontaneous relaxation between these intermediate energy states leads to single photon emission at near IR region around 1.56 eV.

The complete tunability of C_B_ defect, is observed in deep near-IR region and the tunability emission range was listed in Tables 2 and 3. The detailed explanation related to electric field inducement to the C_B_ defected hBN layer was provided in supporting information (Section-3). This electrical tunability of quantum emission around the frontier of optical O-band IR region helps in implementing, efficient quantum communication via optical cable for long range services.

#### Single boron and single nitrogen substitutional with carbons (C_B_C_N_)

This C_B_C_N_ defect is also called carbon dimer. Out of all the carbon related defects, C_B_C_N_ is the only defect which exhibits quantum emission at higher energy region. This Carbon dimer defect is expected to form when the carbon is involved during growth of hBN films.

**Figure 10:**
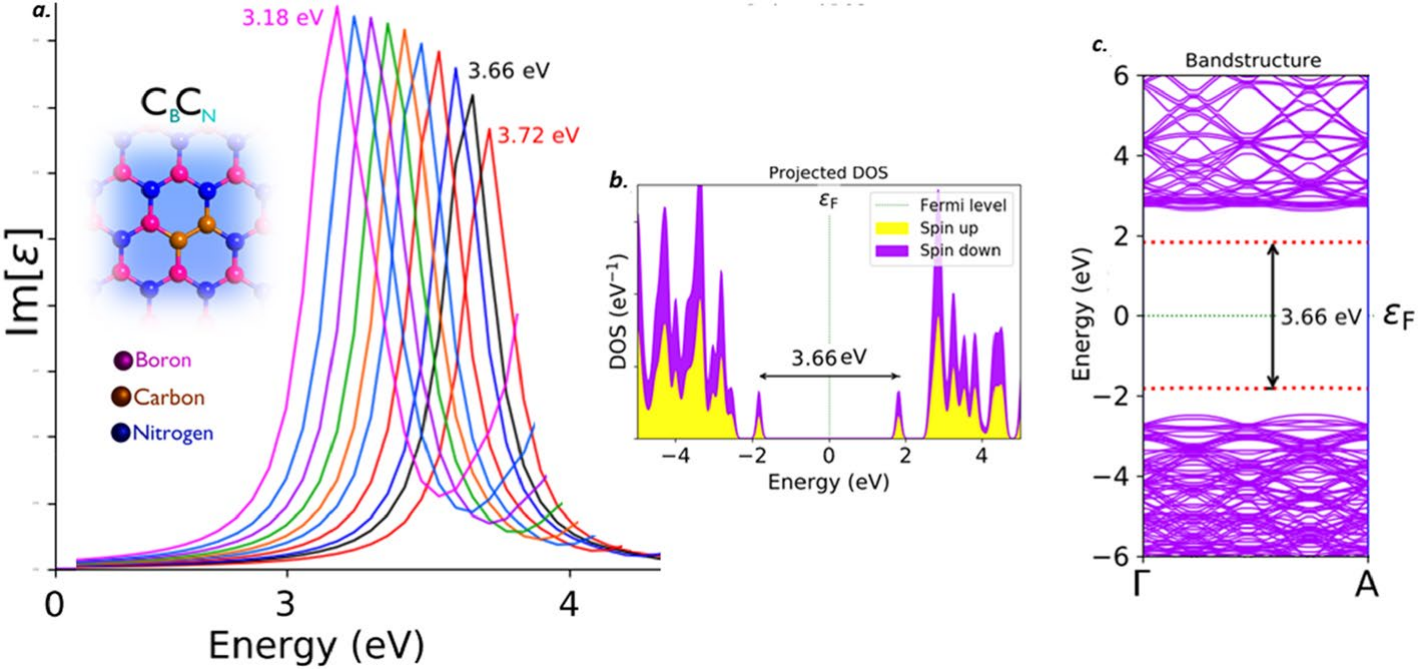
Schematic illustration of C_B_C_N_ defect, its complete optical emission spectrum (tuned by external electric field inducement), corresponding PDOS and band structures. (**a**) The optical emission spectrum with maximum possible tunability due to electric field inducement and the inset cartoon shows the schematic of C_B_C_N_ defect. The ZPL emission without any electric field applied is observed at around 3.66 eV. (**b**) Corresponding PDOS of C_B_C_N_ defect engraved hBN layer without inducing electric field. (**c**) Corresponding energy band structure plot for zero electric field induced, defected hBN layer, whose energy difference is consistent with ZPL and PDOS (Fig. 10(**b**)).

The C_B_C_N_ defect, found to emit the single photons in the UV-A region^34^. This C_B_C_N_ defect exhibits a sharp Lorentzian shaped ZPL emission peak around 3.66 eV (black curve) as shown in Fig. 10a, without any tuning biases inducement like external deformative strain and electric field etc.,. The graphically represented PDOS and similar virtually analytic energy band structure plot of C_B_C_N_ defect under no external static electric field inducement is shown in Fig. 10b and c respectively, which represents the formation of new electron occupied and un-occupied intermediate energy states (formed due to C_B_C_N_ defect) and separated by a Fermi level in between.

The projected intermediate energy states in PDOS, also observed in energy band structure plots (highlighted by red dotted lines). The energy difference between these intermediate energy states in PDOS and band structure are found to be consistent with each other and also consistent with ZPL emission energy. The electronic transition and followed by spontaneous emission between these intermediate energy states, leads to single photon emission in UV-A region at 3.66 eV.

The complete tunability of C_B_C_N_ defect, is observed in deep UV-A region and the tunability emission range was listed in Tables 2 and 3. The detailed explanation related to electric field inducement to the C_B_C_N_ defected hBN layer was provided in supporting information (Section-4). This kind of electrical tuning of quantum emission towards deep UV-A region, efficiently enhances the fabrication of quantum photonic circuits and devices, which finds application in quantum cryptography and quantum information technologies.

#### Carbon dimer complex (C_B_C_N_C_B_C_N_)

Recent first principle DFT calculations have predicted a new carbon based complex defect structure which found to emit single photons. The structure was predicted to be as Carbon dimer complex (C_B_C_N_C_B_C_N_) defect. This predictions was observed to be consistent with experimental results^39^. This defect structure is found to be fabricated by controlled irradiation of electron beam and by using scanning electron microscope. The DFT calculations have also predicted Carbon dimer complex (C_B_C_N_C_B_C_N_) defect in two different structural forms. We have examined the electric field tuning observation on both of these two different structural forms and for analysis purpose, we named them as C_B_C_N_C_B_C_N_ defect (type-1) and C_B_C_N_C_B_C_N_ defect (type-2).

**Figure 11:**
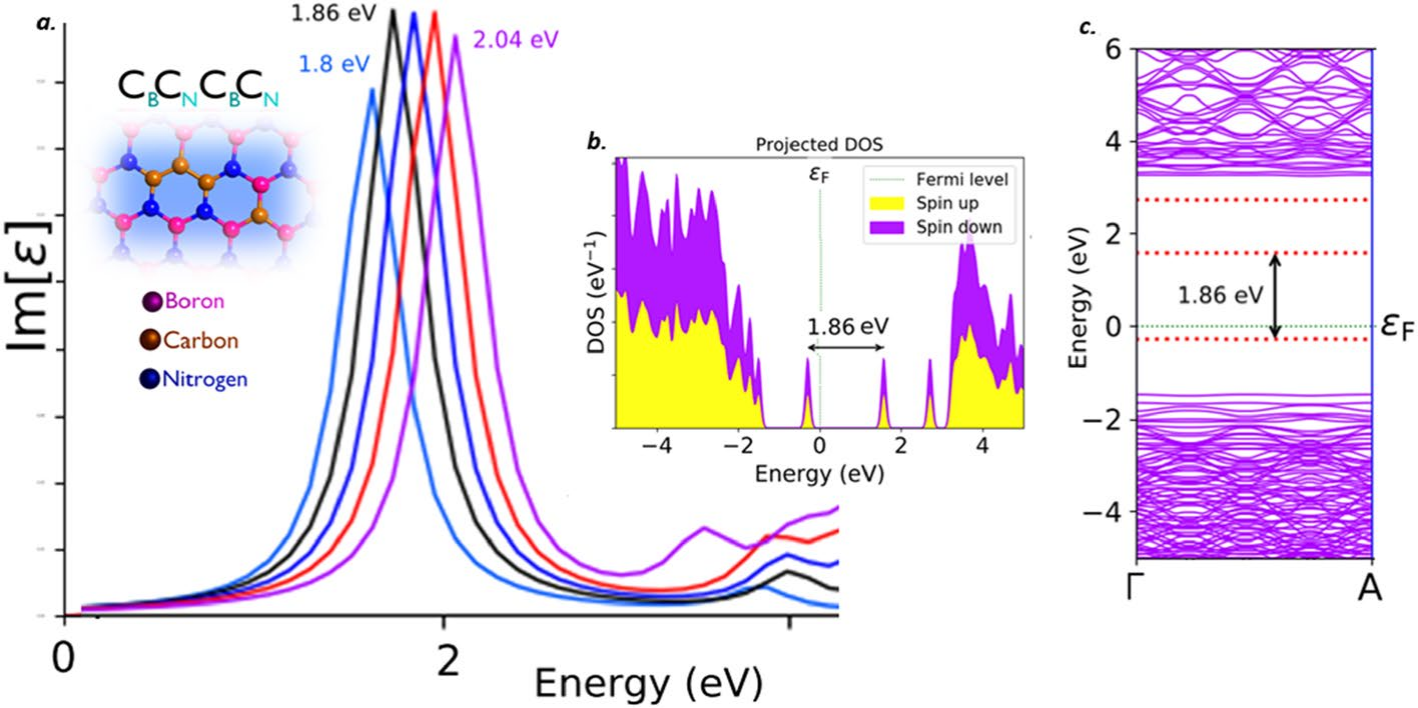
Schematic illustration of C_B_C_N_C_B_C_N_ type-1 defect, its complete optical emission spectrum (tuned by external electric field inducement), corresponding PDOS and band structures. (**a**) The optical emission spectrum with maximum possible tunability due to electric field inducement and the inset cartoon shows the schematic of C_B_C_N_C_B_C_N_ type-1 defect. The ZPL emission without any electric field applied is observed at around 1.86 eV. (**b**) Corresponding PDOS of C_B_C_N_C_B_C_N_ type-1 defect engraved hBN layer without inducing electric field. (**c**) Corresponding energy band structure plot for zero electric field induced, defected hBN layer, whose energy difference is consistent with ZPL and PDOS (Fig. 11(**b**)).

**Figure 12:**
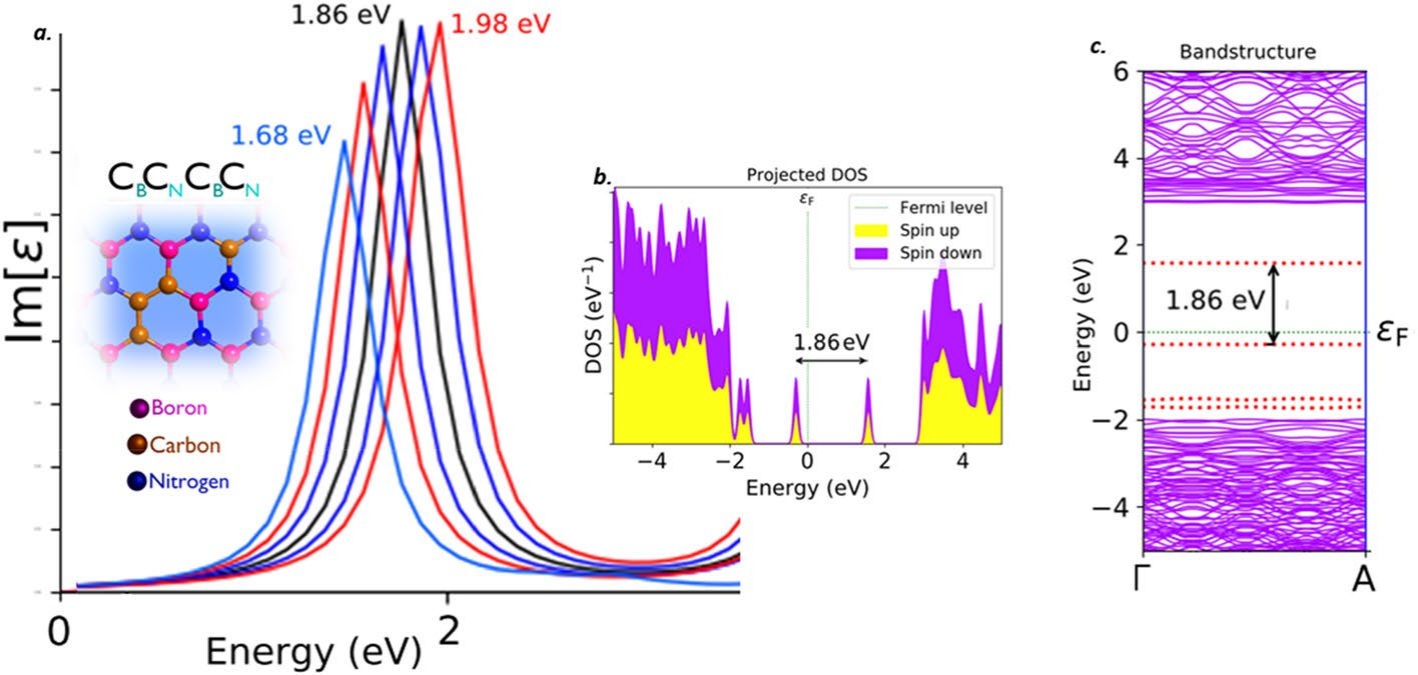
Schematic illustration of C_B_C_N_C_B_C_N_ type-2 defect, its complete optical emission spectrum (tuned by external electric field inducement), corresponding PDOS and band structures. (**a**) The optical emission spectrum with maximum possible tunability due to electric field inducement and the inset cartoon shows the schematic of C_B_C_N_C_B_C_N_ type-2 defect. The ZPL emission without any electric field applied is observed at around 1.86 eV. (**b**) Corresponding PDOS of C_B_C_N_C_B_C_N_ type-2 defect engraved hBN layer without inducing electric field. (**c**) Corresponding energy band structure plot for zero electric field induced, whose energy difference is consistent with ZPL and PDOS (Fig. 12(**b**)).

Both the C_B_C_N_C_B_C_N_ type-1 and type-2 defects, found to emit the single photons in the visible region. This C_B_C_N_C_B_C_N_ type-1 and type-2 defects exhibits a sharp Lorentzian shaped ZPL emission peak around 1.86 eV (black curve) as shown in Figs. 11a and 12a respectively, without any tuning bias inducement like external deformative strain and electric field etc.,. The graphically represented PDOS and similar virtually analytic energy band structure plots of C_B_C_N_C_B_C_N_ type-1 is shown in Fig. 11b and c respectively and for C_B_C_N_C_B_C_N_ type-2 is shown in Fig. 12b and c respectively.

The PDOS and energy band structure plots for both of these defects were obtained under no external static electric field inducement. This PDOS and energy band structures represents the formation of new electron occupied and un-occupied intermediate energy states (formed due to C_B_C_N_C_B_C_N_ (type-1 and type-2) defects) and separated by a Fermi level in between.

The projected intermediate energy states in PDOS, also observed in energy band structure plots (highlighted by red dotted lines). The energy difference between these intermediate energy states in PDOS and band structure are found to be consistent with each other in C_B_C_N_C_B_C_N_ (type-1) defect and also consistent with its corresponding ZPL emission energy. As similar, in C_B_C_N_C_B_C_N_ (type-2) defect, the energy difference between the intermediate energy states in PDOS and band structure are found to be consistent with each other and also consistent with its corresponding ZPL emission energy. The electronic transition and followed by spontaneous emission between these intermediate energy states, leads to single photon emission in visible region for both C_B_C_N_C_B_C_N_ type-1 and type-2 defects at 1.86 eV.

The complete tunability of C_B_C_N_C_B_C_N_ defect complex structures (type-1 and type-2), is observed in visible region and the tunability emission range was listed in Tables 2 and 3. The detailed explanation related to electric field inducement to the C_B_C_N_C_B_C_N_ defected hBN layer was provided in supporting information (Section-5). This kind of tunable quantum emission in visible region, mainly helps in developing small scale quantum devices such as quantum sensors and quantum lamps etc.

#### Stark tuning of passivated point defect structures

After numerous first principle DFT computational predictions, it was clearly stated that the onlypassivated defect structure (among various forms), considered as stable single photon emitter is Boron vacancy with passivated oxygen atoms (VBO_2_).

##### Boron vacancy with passivated oxygen atoms (VBO_2_)

This VBO_2_ defect is with boron mono vacancy and surrounded nitrogen atoms passivated with oxygen. This VBO_2_ defect is formed by Plasma etching with a power of 200 W under a pressure of 180 mTorr of Ar or O_2_ for 2 min at room temperature^30^.

**Figure 13:**
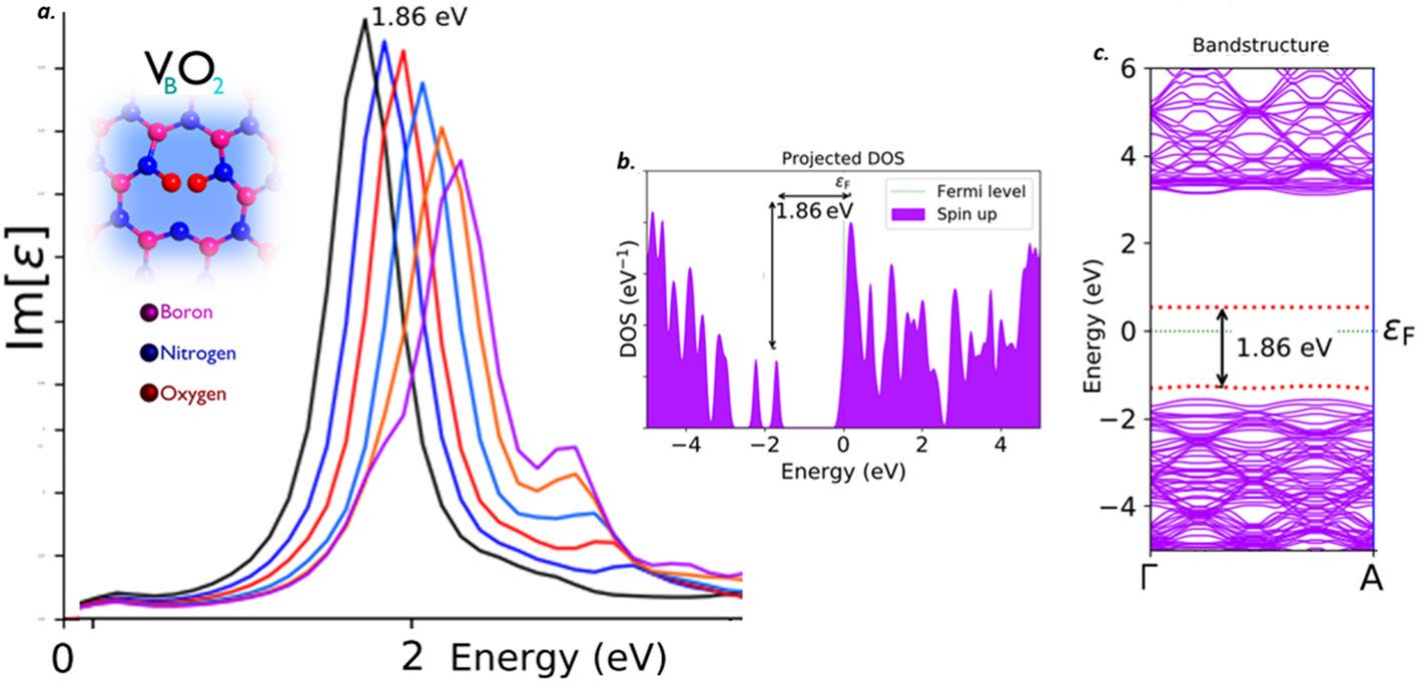
Schematic illustration of VBO_2_ defect, its complete optical emission spectrum (tuned by external electric field inducement), corresponding PDOS and band structures. (**a**) The optical emission spectrum with maximum possible tunability due to electric field inducement and the inset cartoon shows the schematic of VBO_2_ defect. The ZPL emission without any electric field applied is observed at around 1.86 eV (black curve). (**b**) Corresponding PDOS of VBO_2_ defect engraved hBN layer without inducing electric field. (**c**) Corresponding energy band structure plot for zero electric field induced, defected hBN layer, whose energy difference is consistent with ZPL and PDOS (Fig. 13(**b**)).

The VBO_2_ defect, found to emit the single photons at the edge of near-IR region. This VBO_2_ defect exhibits a ZPL emission sharp Lorentzian shaped peak around 1.86 eV (black curve) as shown in Fig. 13a, without any tuning biases inducement like external deformative strain and electric field etc.,. The graphically represented PDOS and similar virtually analytic energy band structure plot of VBO_2_ defect under no external static electric field inducement is shown in Fig. 13b and c respectively, which represents the formation of new electron occupied and un-occupied intermediate energy states (formed due to VBO_2_ defect) and separated by a Fermi level in between.

The projected intermediate energy states in PDOS, also observed in energy band structure plots (highlighted by red dotted lines). The energy difference between these intermediate energy states in PDOS and band structure are found to be consistent with each other and also consistent with ZPL emission energy. The electronic transition and followed by spontaneous emission between these intermediate energy states, leads to single photon emission at the frontier of near-IR region around 1.86 eV.

The complete tunability of VBO_2_ defect is observed towards visible region and the tunability emission range was listed in Tables 2 and 3. The detailed explanation related to electric field inducement to the VBO_2_ defected hBN layer was provided in supporting information (Section-6). This kind of electrical tuning of quantum emission towards visible region, efficiently enhances the fabrication of quantum photonic circuits and quantum processor chips, which finds cooperative in enhancing quantum computing applications.

### Physical interpretations behind quantum light emission wavelengths shift due to external electric field inducement for different defect structures

1. The reason behind wavelength shifts in quantum emission due to electric field inducement is, external electric field tilts the atomic bond angles in defect structure and this enables to create an out-of-plane dipole moment. This dipole creation towards out-of-plane causes tuning of quantum emission.
  This out-of-plane dipole formation phenomena due to electric field inducement as illustrated in Fig. 6 (for N_B_V_N_ and C_B_V_N_ defects) was also observed for other defects i.e. C_B_, C_B_C_N_, C_B_C_N_C_B_C_N_ complex and V_B_O_2_ defects, explored in Figure S8 and S9 respectively.
2. In all the experimental cases, the quantum emission with faint nano range tuning is observed around 600 nm and some of their DFT predictions claims that the emission must be due to N_B_V_N_ defect. As per our DFT computations, this hypothesis must be true, because in our simulation works also we have observed quantum emission tuning around 600 nm for N_B_V_N_ defect as shown in Fig. 5a and the complete tunability is observed throughout the visible region.
  Besides the tuning emission of this N_B_V_N_ defect, we have also observed the tuning towards lower energy region for negative voltage across the gate electrode and for the positive gate voltage, the tuning towards higher energy is observed. This phenomenon is consistent with the experimental case of tuning the quantum emission as observed in ref^21^.
3. The tuning of quantum emission w.r.t the gate voltage polarities is not same for all the point defects structures (i.e., for N_B_V_N_ defect the tuning towards lower energy region is observed for negative gate voltage, which can be in contrast with other defects such as for V_B_ defect, the tuning towards lower energy region is observed for positive gate voltage and vice-versa).
  This occurrence is due to the fact that each luminescent point defect has its own unique characteristics such as different fabrication techniques, defect structure orientations and different energy gaps between intermediate energy states (created due to point defects) and their respective different energy positions etc.
4. For all the defect structures, the increase or decrease in intensities during the energy shifts of ZPL (quantum emission tuning due to electric field inducement) was observed in our simulations. In general, this ZPL intensity represents the emission rate (photons emitted per second**)**^26^ and this increase or decrease in intensity will represent the increase or decrease in emission rate. This observation is consistent with experimental analysis, where discrete change in defect emission intensity is observed for high gate voltage as in ref^20^.
5. The tuning towards O-band IR region is found to observed only for extrinsic defects (such as C_B_ and C_B_V_N_ defects), in which foreign atom impurities such as carbon was involved.
6. It was clearly observed that the intrinsic and passivated point defect structures found to exhibit quantum emission tuning only towards visible and UV regions. Namely N_B_V_N_, VBO_2_ defects towards visible regions, V_B_ defect in complete UV region.
7. Another important hypothesis to mention is about spin preserved transitions of luminescent point defect structures. As explored in earlier material and methodology section, these luminescent point defects structures create an intermediate energy states in between wide bandgap (between valence and conduction bands) of 2-D hBN as shown in ref^26^. During this electron transition from occupied ground state to un-occupied excited state (when excited with enough energy), the single electron found to preserve its own individual spin, (i.e., the electron transmits to the excited state, which possess the same spin type of electron).

This spin type can be either spin-up ↑ or spin-down ↓ and all luminescent point defects (we computationally studied in this research work), found to preserve their spin polarized transitions. As per the information extracted from PDOS, we observed all the defect structures found to exhibit spin-up ↑ transitions.

### Analysis from other higher level approximations

1. Hence our DFT computations provided a valuable information about luminescent point defects and their intrinsic ZPL quantum emission energies, the tunable emission ranges due to external electric field inducements and corresponding PDOS and energy band structures which replicates the intermediate energy states (formed due to luminescent point defect structures) and energy shifts due to electric field inducements. However, our DFT simulations are constrained to address the important aspects of luminescent point defects such as excited states structures^40^ of point defects, spin- orbit and their hyperfine couplings, which is beyond the ability of our present DFT studies^41–45^.
2. Further in-exhaustive computational works (state-of-the-art) such as, GW approximations with Bethe–Salpeter equation (BSE) and other recent methods are necessary to accurately characterize the properties of luminescent point defect structures such as excited states structures, spin–orbit and hyperfine couplings. Moreover, the first principles predictions of luminescent point defect structures, using GW approximations with BSE calculations is a recent advance, hence these simulation executions demand high computational resources, time and cost effective.
3. We have also analyzed some of the earlier GW-BSE approximation calculations of mono vacancy defects such as V_B_ defect, which revealed the presence of electron fully occupied, half-filled and un-occupied energy states in excited state’s structure as in ref^46^. Some of the spin–orbit and hyperfine couplings of degenerate states in V_B_ and C_B_V_N_ defects were also analyzed^47–49^. Notable research findings from GW-BSE approximation calculations is, among the herd of luminescent point defect structures, N_B_V_N_ defect revealed the highest likelihood in correlating its atomic structure with photophysical characteristics^50^.

## Conclusion

To summarize, we perceived the tuning of quantum light emitters engraved in monolayer hBN by external electric field modulation from UV-C to O-band IR region. The major reason predicted for the shifts in quantum emission is that this external electric field creates an out-of-plane dipole moment due to atomic bond angle tilts and bond angle tilts forms a atomic protrude of point defect structure. This tuning range is significant in implementing successful quantum communication applications such as QKD, using fiber optics and free space propagations. By performing constrained DFT computations, we modelled a passive photonic component, in which point defects embedded in hBN monolayer, is incorporated in Metal/graphene/hBN defect structure/graphene/Metal heterostructure. By applying the voltage across metallic gate electrodes of the heterostructure, the electric field is induced at the vicinity of point defects in 2D-hBN defect structure. We extracted the electro-optical parameter responses from energy band structures, associated PDOS plots and ZPL tuning characteristics for each defect structure. The quantum emission tuning towards O-band IR region is obtained by inducing electric field across C_B_V_N_ defect. The C_B_ defect reveals the quantum emission tuning at the frontier of O-band IR region (around 1148 nm). Only V_B_ defect revealed the quantum emission tuning towards solar blind UV-C region. Rest of the defects like N_B_V_N_, C_B_C_N_ and other structures, exhibited quantum emission tuning in visible and UV-A regions. This kind of wide emission tunability with 2D material based passive photonic component promotes the scaling factor and can be easily embedded in the quantum photonic circuits, where single photon emitters need to be seated. Our simulation outcomes may support the effortless tunable quantum emission to the broad wavelength range, with un-challenging feasibility and painless scaling ability, can enhance the efficient implementation of quantum information applications, extensively for QKD. Further highly efficient GW approximation calculations are necessary for in-deep characterization of the luminescent point defects’ excited state’s properties along with spin–orbit and hyperfine couplings properties etc.

### Supplementary Information

Supplementary Information.

## Data Availability

The datasets used and/or analysed during the current study available from the corresponding author on reasonable request.
