# Supplementary material for: Electrical tuning of quantum light emitters in hBN for free space and telecom optical bands
Source: Sci Rep. 2024 Jan 8;14:811. doi: 10.1038/s41598-024-51504-x (PMC10774371; doi:10.1038/s41598-024-51504-x)
Supplement: Supplementary file 1 — Supplementary Information. [file 41598_2024_51504_MOESM1_ESM.pdf]

Supporting information

Electrical Tuning of Quantum Light Emitters in hBN for Free Space and Telecom Optical Bands

Akbar Basha Dhu-al Shaik[1], Penchalaiah Palla[1]* and David Jenkins[2]

[1]Department of Micro and Nanoelectronics, School of Electronics Engineering, Vellore Institute of Technology, Vellore, Tamil Nadu, 632014, India.

[2]School of Engineering, Computing and Mathematics (Faculty of Science and Engineering), University of Plymouth, Plymouth, England, United Kingdom.

*Corresponding author email: penchalaiah.palla@vit.ac.in (official), drpench.palla@gmail.com (personal).

1. Boron mono vacancy (V_B):

By using metallic gates, an electric field was induced, to the V_B defected hBN layer. We observed the quantum emission tuning towards lower energy region for applied positive gate voltage and the quantum emission tuning higher energy region for negative gate voltage. We have induced higher order of gate voltages, in order to examine the V_B defect's maximum tunability. We have observed the tunability towards lower energy region around 3.84 eV (for higher positive gate voltage) and the tunability towards higher energy region around 4.68 eV (for higher negative gate voltage). The complete tunability of V_B defect for the applied electric field was shown in Figure 6(a) and the magnitude of electric field applied for this greater tunability was listed in Table 2.

We also analysed the PDOS and energy band structure plots of electric field induced V_B defected hBN layer. The declination of intermediate energy states was observed in PDOS and energy band structures as shown in Figure S1(a) and S1(b) respectively, for the applied higher order positive gate voltages and so that energy gap between this declined energy states got reduced. This reduced energy gaps in PDOS and energy band structure were consistent with tuned ZPL energy (around 3.84 eV), towards lower energy region. This consistency confirms the quantum emission tuning towards lower energy region, from V_B defect, for the electric field induced and magnitude of electric field applied for tuning towards lower energy region was listed in Table 2.

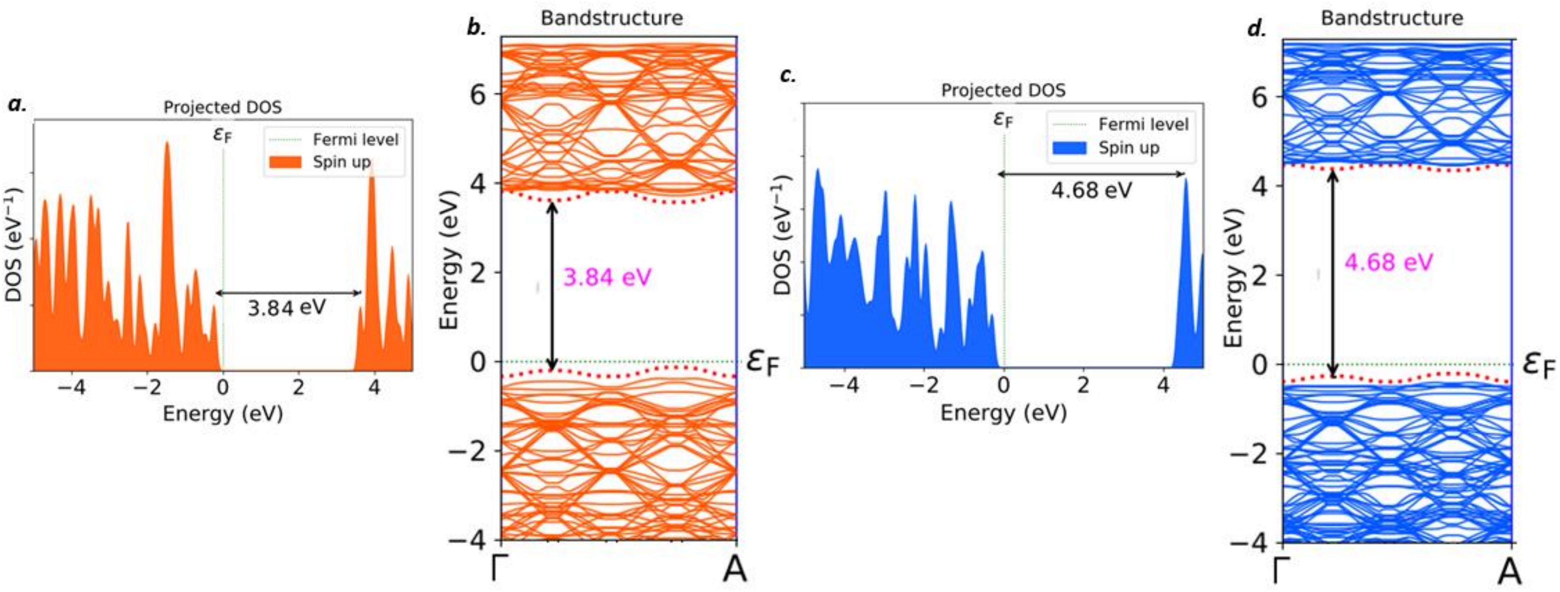

Figure S1: Schematic illustration of V_B defect, its complete optical emission spectrum (tuned by external electric field inducement), corresponding PDOS and band structures. (a) Corresponding PDOS of positive electric field induced V_B defected hBN layer. (b) Corresponding energy band structure plot for positive electric field inducement, whose reduced energy gap between intermediate states is consistent with tuned ZPL and PDOS (Figure S1(a)). (c) Corresponding PDOS of negative electric field induced V_B defected hBN layer. (d) Corresponding energy band structure plot for negative electric field inducement, whose increased energy gap between intermediate states is consistent with tuned ZPL and PDOS (Figure S1(c)). Optical emission spectrum plots were extracted by assigning y-axis to imaginary component of dielectric constant [ε] and x-axis to energy (eV). In all the energy band structure plots intermediate states were highlighted with red dotted lines.

Similarly, for higher order negative gate voltages, the energy gaps between this intermediate energy states were incremented, in PDOS and band structure plots as shown in Figure S1(c) and S1(d) respectively. This increased energy gap values of PDOS and energy band structure were consistent with tuned ZPL energy (around 4.68 eV), towards higher energy region. This consistency confirms the quantum emission tuning towards higher energy region, for the applied negative electric field, from V_B defect.

2. **Nitrogen mono vacancy with carbon-interstitial (C_BV_N):**

Now an electric field was induced, to the C_BV_N defect engraved hBN layer, by using metallic gates. Due to this, we observed the quantum emission tuning towards lower energy region for applied positive gate voltage and for negative gate voltage, the quantum emission tuning higher energy region was observed. In order to inspect the C_BV_N defect's maximum tunability, we have induced higher order of gate voltages.

We have observed the tunability towards lower energy region around 0.96 eV (for higher positive gate voltage) and the tunability towards higher energy region around 1.5 eV (for higher negative gate voltage). The

complete tunability of C_BV_N defect for the applied electric field was shown in Figure 8(a) and the magnitude of electric field applied for this greater tunability was listed in Table 2.

We also inspected the PDOS and energy band structure plots of electric field induced C_BV_N defected hBN layer. The diminishing of intermediate energy states was observed in PDOS and energy band diagrams as shown in Figure S2(a) and S2(b) respectively, for the applied higher order positive gate voltages and so that energy gap between this diminished energy states got decreased. This decreased energy gaps in PDOS and energy band diagram were consistent with tuned ZPL energy (around 0.96 eV), towards lower energy region. This consistency confirms the quantum emission tuning towards lower energy region, from C_BV_N defect, for the electric field induced and magnitude of electric field applied for tuning towards lower energy region was listed in Table 2.

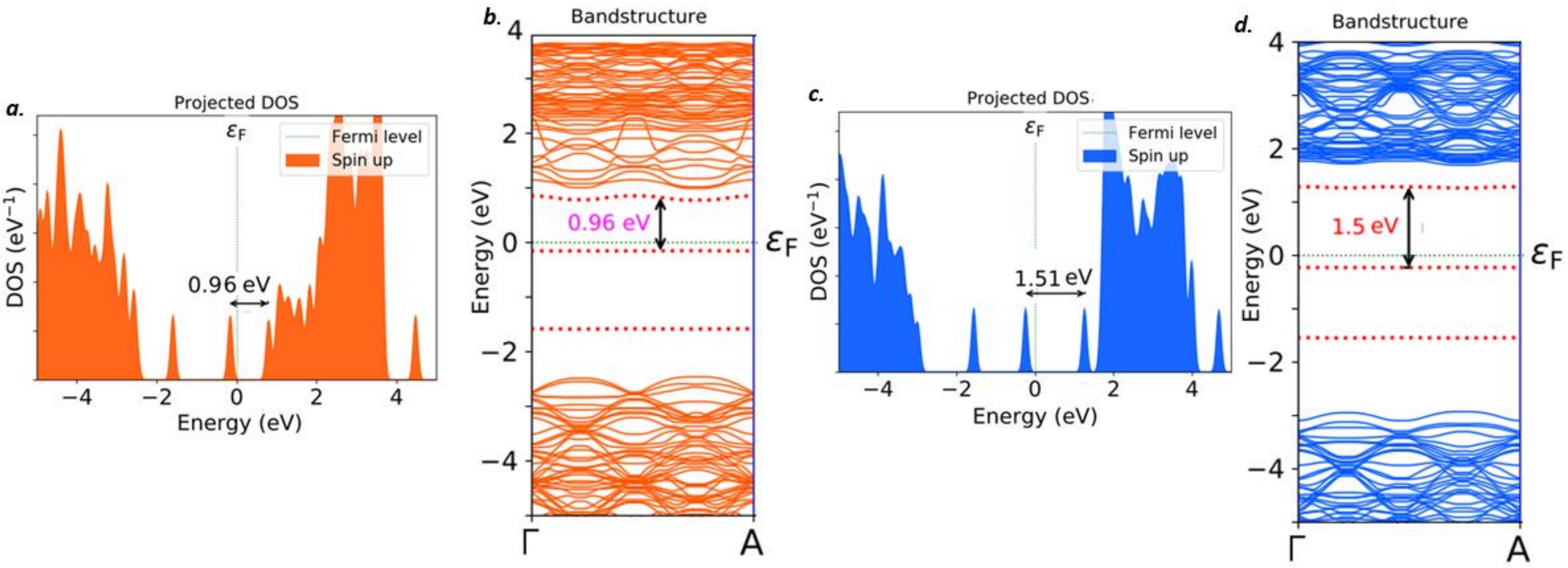

Figure S2: Schematic illustration of C_BV_N defect, its complete optical emission spectrum (tuned by external electric field inducement), corresponding PDOS and band structures. (a) Corresponding PDOS of positive electric field induced C_BV_N defected hBN layer. (b) Corresponding energy band structure plot for positive electric field inducement, whose reduced energy gap between intermediate states is consistent with tuned ZPL and PDOS (Figure S2(a)). (b) Corresponding PDOS of negative electric field induced C_BV_N defected hBN layer. (d) Corresponding energy band structure plot for negative electric field inducement, whose increased energy gap between intermediate states is consistent with tuned ZPL and PDOS (Figure S2(c)). Optical emission spectrum plots were extracted by assigning y-axis to imaginary component of dielectric constant [ε] and x-axis to energy (eV). In all the energy band structure plots intermediate states were highlighted with red dotted line.

As similar, the energy gaps between this intermediate energy states were increased, in PDOS and band diagrams plots as shown in Figure S2(c) and S2(d) respectively, for higher order negative gate voltages. This increased energy gap values of PDOS and energy band diagrams were consistent with tuned ZPL energy (around 1.5 eV), towards higher energy region. This consistency confirms the quantum emission tuning towards higher energy region, for the applied positive electric field, from C_BV_N defect.

3. **Single boron substitutional with carbon (C_B):**

In order to examine the defect's extreme tunability, we have induced higher order gate voltages and we observed the tunability towards lower energy region around 1.08 eV (for higher negative gate voltage) and the tunability towards higher energy region around 1.8 eV (for higher positive gate voltage). The complete tunability of C_B defect for the applied electric field was shown in Figure 9(a) and the magnitude of electric field applied for this greater tunability was listed in Table 2.

We also examined the PDOS and energy band structure plots of electric field induced defective hBN layers. The decrease in energy gaps in PDOS, is due to de-escalation of intermediate energy states for the higher order negative gate voltage was shown in Figure S3(a) and the similar behaviour was observed in band structure plots as shown in Figure S3(b). The diminished energy gap in PDOS and band structure plots were consistent with tuned ZPL energy (around 1.08 eV), towards lower energy region. This consistency confirms the quantum emission tuning towards lower energy region, from C_B defect, for the electric field induced and magnitude of electric field applied for tuning towards lower energy region was listed in Table 2.

Similarly, for higher order positive gate voltages, the energy gaps between this intermediate energy states were inclined and this inclination of energy gaps in PDOS and band structure plots as shown in Figure S3(c) and S3(d) respectively. The increased energy gap values of PDOS and energy band structure were consistent with tuned ZPL energy (around 1.8 eV), towards higher energy region. This consistency assures the quantum emission tuning towards higher energy region, from C_B defect, for the applied electric field.

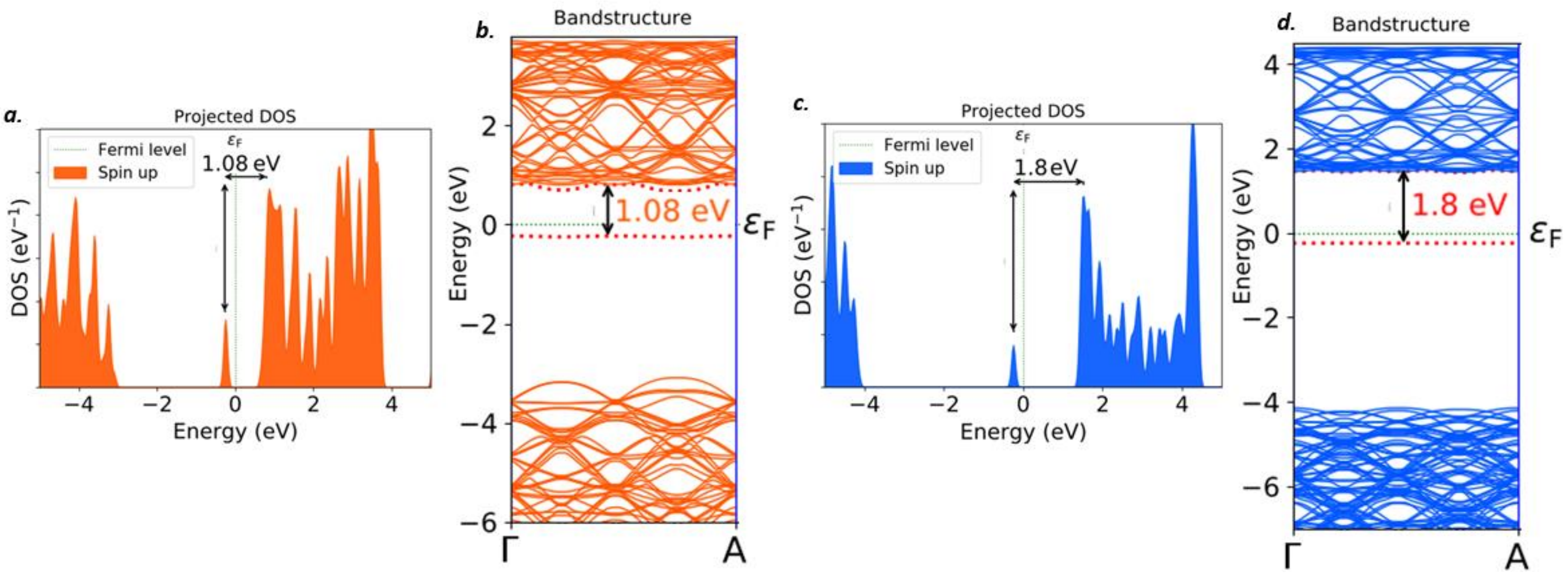

Figure S3: Schematic illustration of C_B defect, its complete optical emission spectrum (tuned by external electric field inducement), corresponding PDOS and band structures. (a) Corresponding PDOS of negative electric field induced C_B defected hBN layer. (b) Corresponding energy band structure plot for negative electric field inducement, whose reduced energy gap between intermediate states is consistent with tuned ZPL and PDOS (Figure S3(a)). (c) Corresponding PDOS of positive electric field induced C_B defected hBN layer. (d) Corresponding energy band structure plot for positive electric field inducement, whose increased energy gap between intermediate states is consistent with tuned ZPL and PDOS (Figure S3(c)). Optical emission spectrum plots were extracted by assigning y-axis to imaginary component of dielectric constant [ε] and x-axis to energy (eV). In all the energy band structure plots intermediate states were highlighted with red dotted lines.

4. **Single boron and single nitrogen substitutional with carbons (C_BC_N):** Now an electric field was induced, to the C_BC_N defect engraved hBN layer, by using metallic gates. Due to this, we observed the quantum emission tuning towards lower energy region for applied negative gate voltage and for positive gate voltage, the quantum emission tuning higher energy region was observed. In order to inspect the C_BC_N defect's maximum tunability, we have induced higher order of gate voltages. We have observed the tunability towards lower energy region around 3.18 eV (for higher negative gate voltage) and the tunability towards higher energy region around 3.72 eV (for higher positive gate voltage). The complete tunability of C_BC_N defect for the applied electric field was shown in Figure 10(a) and the magnitude of electric field applied for this greater tunability was listed in Table 2.

   We also inspected the PDOS and energy band structure plots of electric field induced C_BC_N defected hBN layer. The diminishing of intermediate energy states was observed in PDOS and energy band diagrams as shown in Figure S4(a) and S4(b) respectively, for the applied

higher order negative gate voltages and so that energy gap between this diminished energy states got decreased. This decreased energy gaps in PDOS and energy band diagram were consistent with tuned ZPL energy (around 3.18 eV), towards lower energy region. This consistency confirms the quantum emission tuning towards lower energy region, from C_BC_N defect, for the electric field induced and magnitude of electric field applied for tuning towards lower energy region was listed in Table 2.

As similar, the energy gaps between this intermediate energy states were increased, in PDOS and band diagrams plots as shown in Figure S4(c) and S4(d) respectively, for higher order positive gate voltages. This increased energy gap values of PDOS and energy band diagrams were consistent with tuned ZPL energy (around 3.72 eV), towards higher energy region. This consistency confirms the quantum emission tuning towards higher energy region, for the applied positive electric field, from C_BC_N defect.

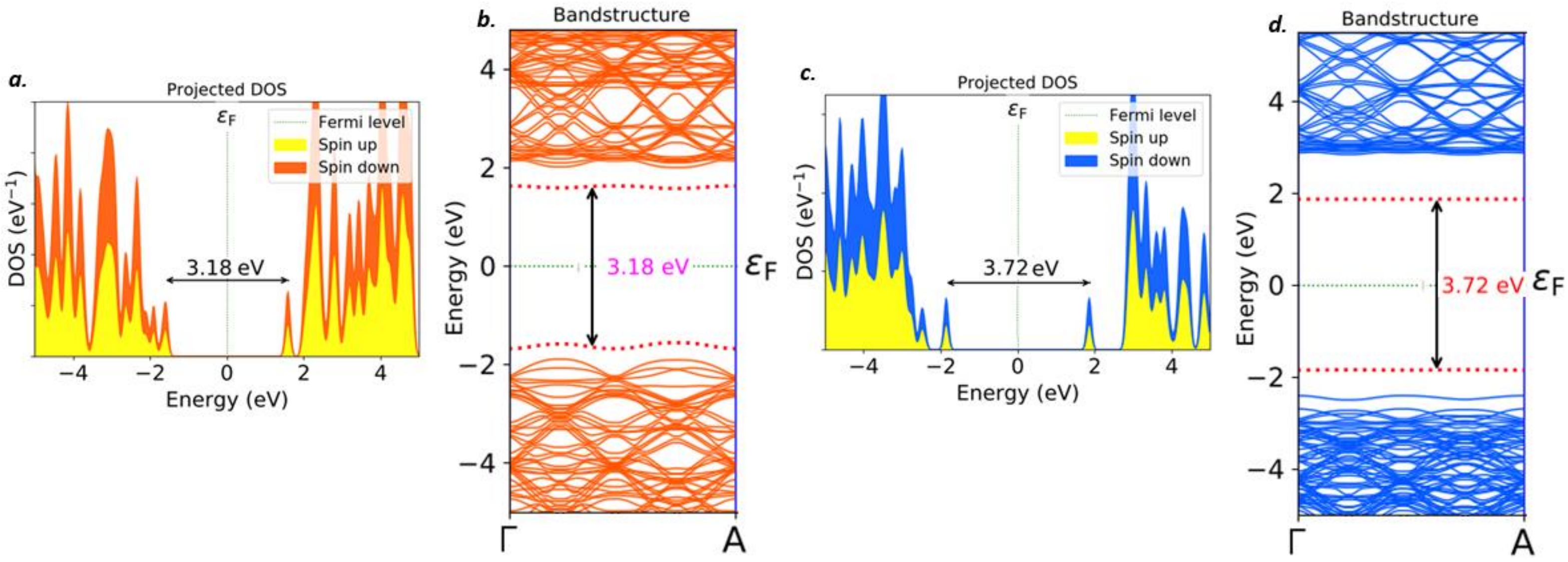

Figure S4: Schematic illustration of C_BC_N defect, its complete optical emission spectrum (tuned by external electric field inducement), corresponding PDOS and band structures. (a) Corresponding PDOS of negative electric field induced C_BC_N defected hBN layer. (b) Corresponding energy band structure plot for negative electric field inducement, whose reduced energy gap between intermediate states is consistent with tuned ZPL and PDOS (Figure S4(a)). (c) Corresponding PDOS of positive electric field induced C_BC_N defected hBN layer. (d) Corresponding energy band structure plot for positive electric field inducement, whose increased energy gap between intermediate states is consistent with tuned ZPL and PDOS (Figure S4(c)). Optical emission spectrum plots were extracted by assigning y-axis to imaginary component of dielectric constant [ε] and x-axis to energy (eV). In all the energy band structure plots intermediate states were highlighted with red dotted lines.

5. **Carbon dimer complex ($C_BC_NC_BC_N$)**:

Now we have induced electric field through metallic gates to both the $C_BC_NC_BC_N$ type-1 and type-2 defects engraved hBN layers respectively. By applying a positive gate voltage at the metallic electrode, we observed the quantum energy tuning towards lower energy region, for both the $C_BC_NC_BC_N$ type-1 and type-2 defect structures and vice versa for negative gate voltage, the quantum energy tuning towards higher energy region were observed.

In order to examine the defect's extreme tunability, we have induced higher order gate voltages and we observed the tunability towards lower energy region around 1.8 eV and 1.68 eV (for higher positive gate voltages) for $C_BC_NC_BC_N$ type-1 and type-2 defect structures respectively. For higher negative gate voltages, tunability towards higher energy region around 2.04 eV and 1.98 eV was observed for $C_BC_NC_BC_N$ type-1 and type-2 defect structures respectively. The complete tunability of $C_BC_NC_BC_N$ type-1 and type-2 defect structures for the applied electric field was shown in Figure 11(a) and 12(a) respectively and the magnitude of electric field applied for this greater tunability was listed in Table 2.

We also inspected the PDOS and energy band structure plots of electric field induced $C_BC_NC_BC_N$ type-1 and type-2 structures defected hBN layers. The diminishing of intermediate energy states was observed in PDOS and energy band diagrams as shown in Figure S5(a) and S5(b) respectively, for the applied higher order positive gate voltage, for $C_BC_NC_BC_N$ type-1 defect structure. As similar, the diminishing of intermediate energy states was also observed in PDOS and energy band diagrams of $C_BC_NC_BC_N$ type-2 defect structure as shown in Figure S6 (a) and S6(b) respectively, for the applied higher order positive gate voltage.

Hence, the energy gap between this diminished energy states got decreased for both the $C_BC_NC_BC_N$ type-1 and type-2 defect structures and this decreased energy gaps in PDOS and energy band diagrams were consistent with tuned ZPL energy (around 1.8 eV and 1.68 eV respectively), towards lower energy region. This consistency confirms the quantum emission tuning towards lower energy region, from $C_BC_NC_BC_N$ type-1 and type-2 defects, for the positive electric field induced and magnitude of electric field applied for tuning towards lower energy region was listed in Table 2.

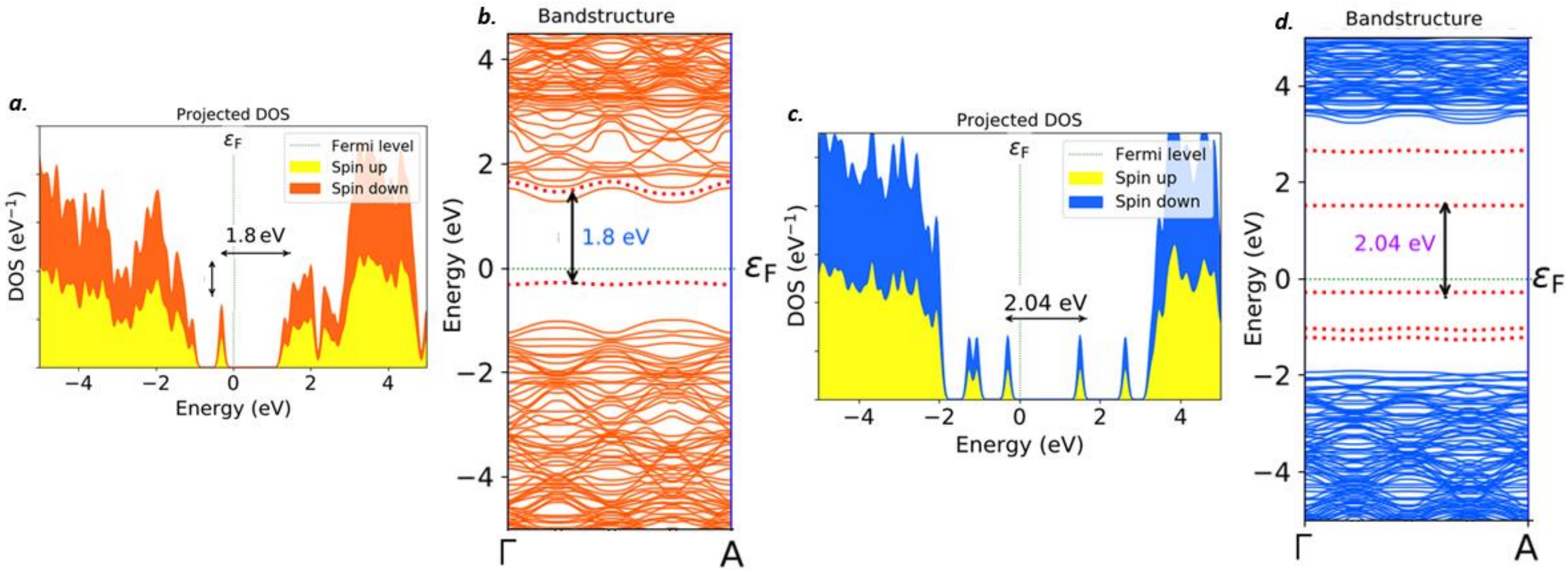

Figure S5: Schematic illustration of $C_BC_NC_BC_N$ type-1 defect, its complete optical emission spectrum (tuned by external electric field inducement), corresponding PDOS and band structures. (a) Corresponding PDOS of positive electric field induced $C_BC_NC_BC_N$ type-1 defected hBN layer. (b) Corresponding energy band structure plot for positive electric field inducement, whose reduced energy gap between intermediate states is consistent with tuned ZPL and PDOS (Figure S5(a)). (c) Corresponding PDOS of negative electric field induced $C_BC_NC_BC_N$ type-1 defected hBN layer. (d) Corresponding energy band structure plot for negative electric field inducement, whose increased energy gap between intermediate states is consistent with tuned ZPL and PDOS (Figure S5(c)). Optical emission spectrum plots were extracted by assigning y-axis to imaginary component of dielectric constant [ε] and x-axis to energy (eV). In all the energy band structure plots intermediate states were highlighted with red dotted lines.

Similarly, the energy gaps between this intermediate energy states were increased, in PDOS and band diagrams plots as shown in Figure S5(c) and S5(d), for $C_BC_NC_BC_N$ type-1 defect and for $C_BC_NC_BC_N$ type-2 defect, the similar increased intermediate energy gaps was observed in PDOS and energy band structures as shown in Figure S6(c) and S6(d) respectively, for higher order negative gate voltages. This increased energy gap values of PDOS and energy band diagrams were consistent with tuned ZPL energies of $C_BC_NC_BC_N$ type-1 and type-2 defects (around 2.04 eV and 1.98 eV respectively), towards higher energy region. This consistency confirms the quantum emission tuning towards higher energy region from $C_BC_NC_BC_N$ type-1 and type-2 defect structures, for the applied negative electric field.

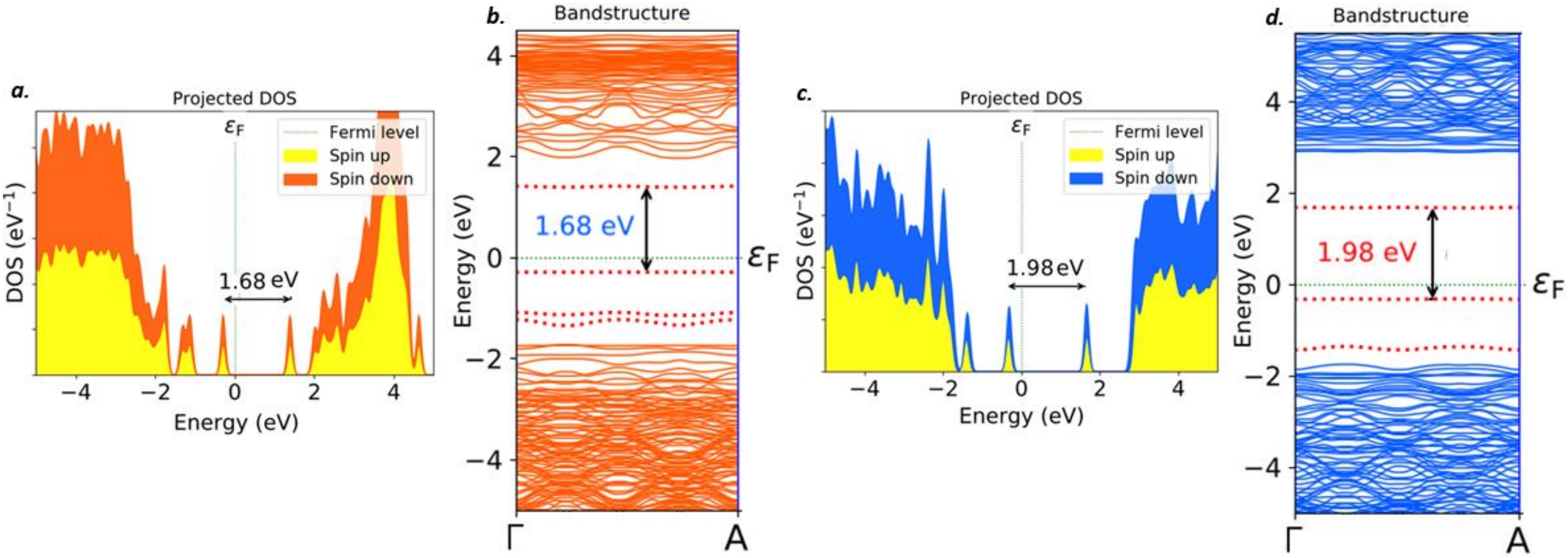

Figure S6: Schematic illustration of $C_BC_NC_BC_N$ type-2 defect, its complete optical emission spectrum (tuned by external electric field inducement), corresponding PDOS and band structures. (a) Corresponding PDOS of positive electric field induced $C_BC_NC_BC_N$ type-2 defected hBN layer. (b) Corresponding energy band structure plot for positive electric field inducement, whose reduced energy gap between intermediate states is consistent with tuned ZPL and PDOS (Figure S6(a)). (c) Corresponding PDOS of negative electric field induced $C_BC_NC_BC_N$ type-2 defected hBN layer. (d) Corresponding energy band structure plot for negative electric field inducement, whose increased energy gap between intermediate states is consistent with tuned ZPL and PDOS (Figure S6(c)). Optical emission spectrum plots were extracted by assigning y-axis to imaginary component of dielectric constant [ε] and x-axis to energy (eV). In all the energy band structure plots intermediate states were highlighted with red dotted lines.

6. Boron vacancy with passivated oxygen atoms (VBO_2):

Now an electric field was induced, to the VBO_2 defect engraved hBN layer, by using metallic gates. Exceptionally for VBO_2 defect, we have only observed the quantum emission tuning towards higher energy region irrespective of gate voltage polarities (i.e. for both applied positive and for negative gate voltages). In order to inspect the VBO_2 defect's maximum tunability, we have induced higher order of positive gate voltages. We have observed the tunability towards higher energy region around 2.16 eV (for higher positive gate voltage).

The complete tunability of VBO_2 defect for the applied electric field was shown in Figure 13(a) and the magnitude of electric field applied for this greater tunability was listed in Table 2.

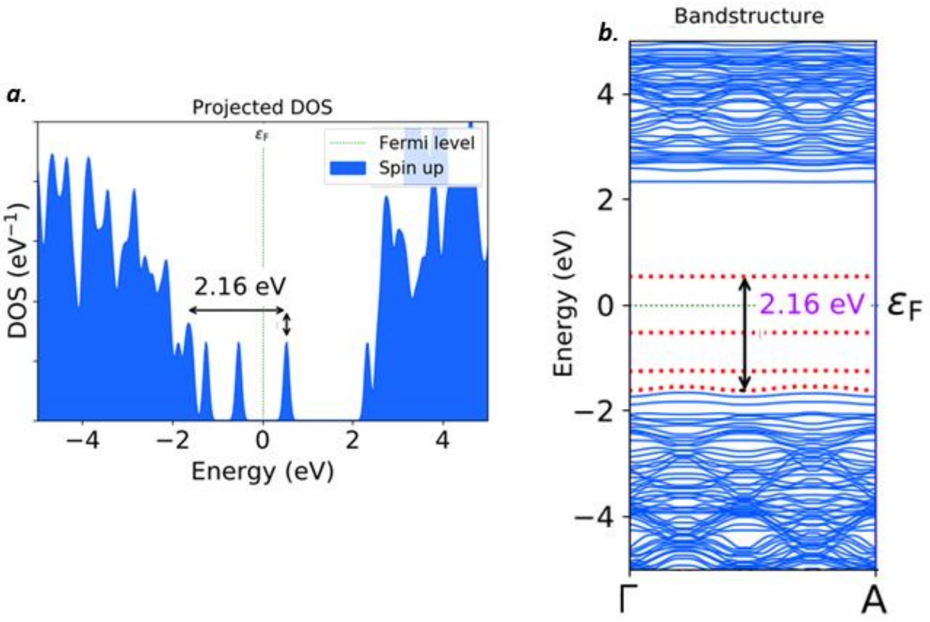

Figure S7: Schematic illustration of VBO_2 defect, its complete optical emission spectrum (tuned by external electric field inducement), corresponding PDOS and band structures. (a) Corresponding PDOS of positive electric field induced VBO_2 defected hBN layer. (b) Corresponding energy band structure plot for positive electric field inducement, whose increased energy gap between intermediate states is consistent with tuned ZPL and PDOS (Figure S7(b)). Optical emission spectrum plots were extracted by assigning y-axis to imaginary component of dielectric constant [ε] and x-axis to energy (eV). In all the energy band structure plots intermediate states were highlighted with red dotted lines.

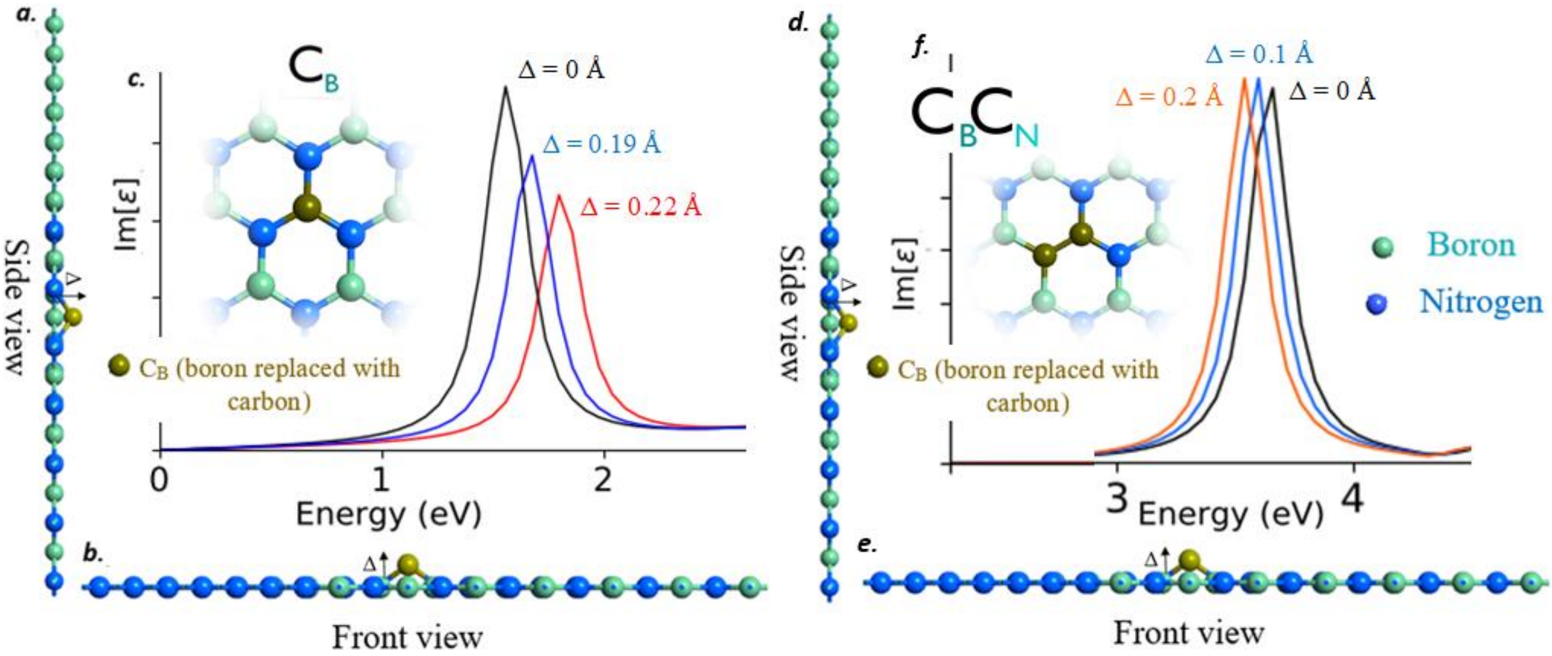

Figure S8: Side and front view of C_B and C_BC_N defects engraved monolayer hBN and corresponding shifts in quantum emission. (a, b) side and front view of monolayer hBN in which C_B defect is engraved. C_B chunk is protruded towards out-of-the crystal plane due to atomic bond angle tilts. (c) Corresponding shifts in quantum emission for different atomic displacements. This different atomic displacements (Δ) were created due to different atomic bond angle tilts. Inset of the Figure (c) shows the C_B defect structure in which C_B segment is popped out. (d, e) side and front view of monolayer hBN in which C_BC_N defect is engraved. C_N chunk from C_BC_N defect is protruded towards out-of-the crystal plane due to atomic bond angle tilts. (f) Corresponding shifts in quantum emission for different atomic displacements. This different atomic displacements were created due to different atomic bond angle tilts. Inset of the Figure (f) shows the C_BC_N defect structure in which C_N segment is popped out.

We also inspected the PDOS and energy band structure plots of electric field induced VBO_2 defected hBN layer. The increment of intermediate energy states was observed in PDOS and energy band diagrams as shown in Figure S7(a) and S7(b) respectively, for the applied higher order positive gate voltages and so that energy gap between this incremented energy states got increased. This increased energy gaps in PDOS and energy band diagram were consistent with tuned ZPL energy (around 2.16 eV), towards higher energy region.

This consistency confirms the quantum emission tuning towards higher energy region, from VBO_2 defect, for the positive electric field induced and magnitude of electric field applied for tuning towards higher energy region was listed in Table 2.

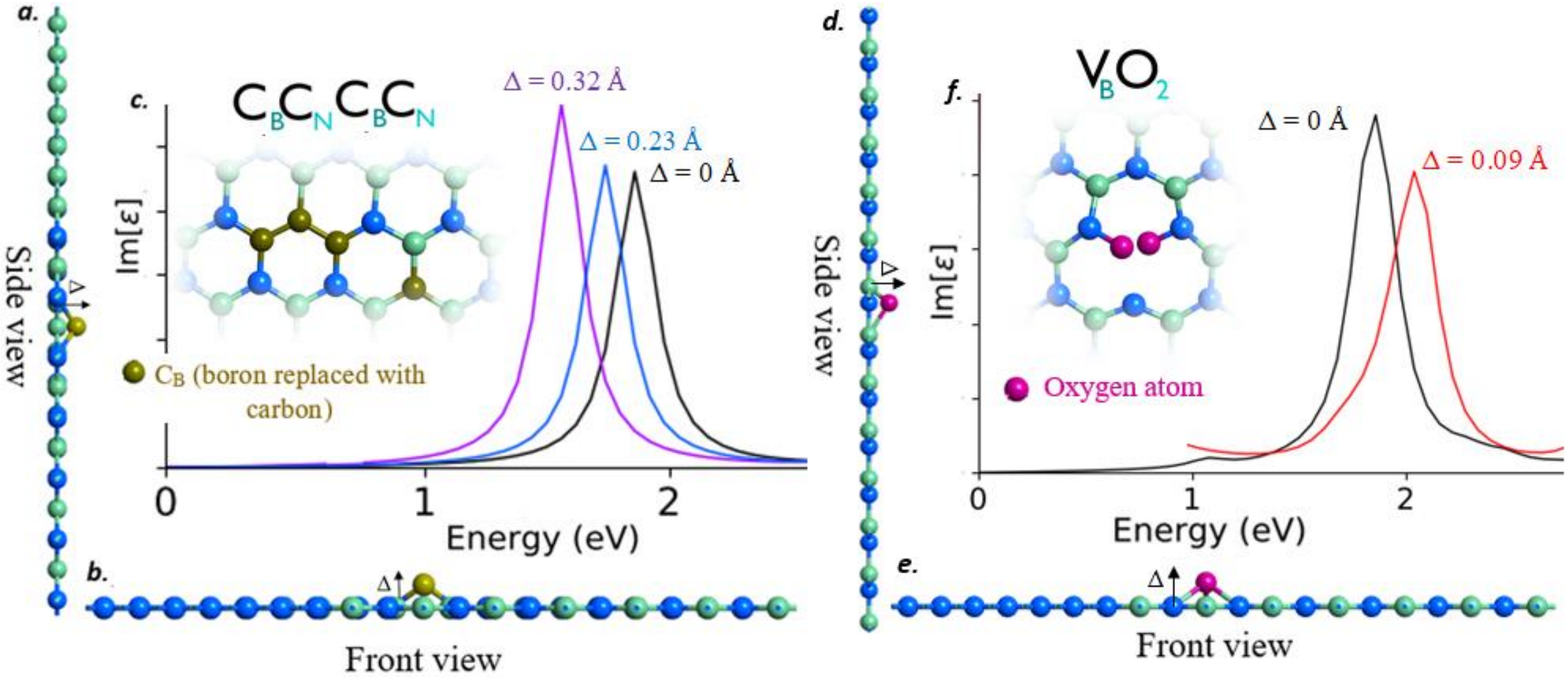

Figure S9: Side and front view of $C_BC_NC_BC_N$ complex and VBO_2 defects engraved monolayer hBN and corresponding shifts in quantum emission. (a, b) side and front view of monolayer hBN in which $C_BC_NC_BC_N$ defect is engraved. A portion of $C_BC_NC_BC_N$ defect is protruded towards out-of-the crystal plane due to atomic bond angle tilts. (c) Corresponding shifts in quantum emission for different atomic displacements. This different atomic displacements (Δ) were created due to different atomic bond angle tilts. Inset of the Figure (c) shows the $C_BC_NC_BC_N$ defect structure in which a portion of defect structure is popped out. (d, e) side and front view of monolayer hBN in which VBO_2 defect is engraved. A chunk of VBO_2 defect is protruded towards out-of-the crystal plane due to atomic bond angle tilts. (f) Corresponding shifts in quantum emission for different atomic displacements. This different atomic displacements were created due to different atomic bond angle tilts. Inset of the Figure (f) shows the VBO_2 defect structure in which a segment of defect structure is popped out.
